# ClBeclin1 Positively Regulates Citrus Defence Against Citrus Yellow Vein Clearing Virus Through Mediating Autophagy‐Dependent Degradation of ClAPX1

**DOI:** 10.1111/mpp.70041

**Published:** 2024-12-10

**Authors:** Jiajun Wang, Ling Yu, Jinfa Zhao, Shimin Fu, Yalin Mei, Binghai Lou, Yan Zhou

**Affiliations:** ^1^ Integrative Science Center of Germplasm Creation in Western China (CHONGQING) Science City Southwest University/National Citrus Engineering and Technology Research Center, Citrus Research Institute, Southwest University Chongqing China; ^2^ Guangxi Key Laboratory of Germplasm Innovation and Utilization of Specialty Commercial Crops in North Guangxi Guangxi Academy of Specialty Crops Guilin Guangxi China

**Keywords:** autophagy, *Citrus yellow vein clearing virus*, ClAPX1 protein, ClBeclin1 protein, jasmonic acid (JA), reactive oxygen species (ROS)

## Abstract

Autophagy, one of the most widespread and highly conserved protein degradation systems in eukaryotic cells, plays an important role in plant growth, development and stress response. Beclin 1 is a core component of the phosphatidylinositol 3‐kinase (PI3K) autophagy complex and positively regulates plant immunity against viruses. The upregulation of Eureka lemon *ClBeclin1* was observed in response to citrus yellow vein clearing virus (CYVCV) infection. However, the function of *ClBeclin1* and the underlying mechanism during CYVCV colonisation remain unclear. Here, the resistance evaluation of the overexpression and silencing of *ClBeclin1* in Eureka lemon hairy roots revealed it as a positive regulator of citrus immunity against CYVCV. Transcriptomic profiling and metabolic analyses along with genetic evidence implied that the overexpression of *ClBeclin1* positively triggered reactive oxygen species (ROS)‐ and jasmonic acid (JA)‐mediated immunity in citrus. The accumulation of ROS and JA contents was attributed to the autophagic degradation of the ROS scavenger ClAPX1 via ClBeclin1 overexpression. Exogenous application of either H_2_O_2_ or JA significantly reduced CYVCV colonisation and vein‐clearing symptoms on the host. Collectively, our findings indicate that *ClBeclin1* activation contributes to citrus immunity against CYVCV through triggering ROS‐ and JA‐mediated defence responses, and the accumulation of ROS and JA resulted from the autophagic degradation of ClAPX1 by ClBeclin1.

## Graphical abstract

During citrus yellow vein clearing virus infection, ClBeclin1 targets ClAPX1 for degradation through autophagy pathway to promote reactive oxygen species and jasmonic acid production, enhancing disease resistance in Eureka lemon.

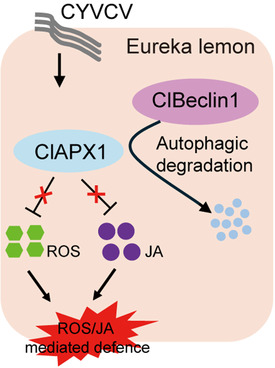

## 1 Introduction

Citrus yellow vein clearing virus (CYVCV) has become an important threat to the citrus industry worldwide. The virus typically infects a wide range of citrus species, with lemon ( *Citrus limon* ) and sour orange (*C. aurantium*) exhibiting pronounced leaf distortion and distinct yellow vein clearing (Loconsole et al. 2012; Chen et al. 2014). CYVCV belongs to the genus *Mandarivirus* in the family *Alphafexiviridae* (Zerbini et al. 2023). The viral genome is approximately 7.5 kb and contains six predicted open reading frames (ORFs) (Loconsole et al. 2012). ORF5 encodes the viral coat protein (CP), which has gene silencing suppressor activity and is positively correlated with the severity of symptoms (Rehman et al. 2019; Bin et al. 2023). Although the lemon 40S ribosomal protein ClRPS9‐2 and zinc finger protein ClDOF3.4 can activate the expression of salicylic acid (SA) and reactive oxygen species (ROS) pathway genes, which inhibits the accumulation of CYVCV and the development of symptoms in plants, the plant defence response to CYVCV infection is poorly understood (Zeng et al. 2023; Liao et al. 2024).

Cellular autophagy is a highly conserved mechanism in eukaryotes that degrades misfolded proteins and damaged organelles, allowing nutrients to be recycled (Zhuang et al. 2015; Qi, Xia, and Xiao 2021; Rehman et al. 2021). In the process of plant immunity, autophagy‐related genes (ATGs) can target viral proteins for autophagic degradation, thereby limiting viral infection (Hafrén et al. 2018; Li et al. 2018). Autophagy also regulates reactive oxygen species (ROS) or plant hormones to be involved in disease resistance responses (Hofius et al. 2009; Yoshimoto et al. 2009). The mammalian gene *Beclin1* is a direct homologue of *ATG6* in plants/yeast and plays a crucial role in regulating membrane trafficking, autophagy, growth, as well as response to stresses (Seay, Patel, and Dinesh‐Kumar 2006; Fujiki, Yoshimoto, and Ohsumi 2007; Qin et al. 2007; Singh et al. 2024). The glycoprotein encoded by *Rice stripe mosaic cytorhabdovirus* interacts with OsSnRK1B, enhancing the kinase activity of OsSnRK1B on OsATG6b to initiate autophagy, targeting the degradation of viral glycoproteins, and limiting virus infection (Huang et al. 2024). It has been also shown that *Beclin1* acts as a positive regulator of pathogen‐induced programmed cell death (PCD) in *N*‐gene‐mediated plant immunity (Liu et al. 2005; Patel and Dinesh‐Kumar 2008). Further research has found that the plant cell death inhibitor *Bax* Inhibitor‐1 (BI‐1) interacts with Beclin1 to promote autophagy and suppress cell death (Xu et al. 2017).

ROS serve as one of the effective defence strategies in plants and play an important role in pathogen stress (Baxter, Mittler, and Suzuki 2014; Wu et al. 2017; Zhu et al. 2020, 2021). However, excessive accumulation of ROS is detrimental to plants. Therefore, plants have evolved a series of antioxidant mechanisms to prevent the accumulation of ROS. As a key enzyme in the ROS scavenging system, ascorbate peroxidase (APX) plays a critical role in oxidative stress responses to both biotic and abiotic stresses. Usually, *APX* plays a negative regulatory role in pathogens that cause cell death (Gou et al. 2015; Jiang et al. 2016; Zhang et al. 2022), whereas it plays a positive regulatory role in compatible plant pathogens (Du et al. 2023). Recent research has shown that APX enzyme activity was significantly increased in CYVCV‐infected Eureka lemon (Zhang, Song, et al. 2023). Wang et al. (2023) further discovered that ClAPX1 interacts with the CP of CYVCV and promotes virus infection by inhibiting the accumulation of H_2_O_2_ and the expression of jasmonic acid (JA) signalling pathway genes.

However, currently whether ClBeclin1 is involved in Eureka lemon resistance to CYVCV by regulation of ClAPXs is unknown. In this study, we have demonstrated the role of ClBeclin1 and its target ClAPX1 in regulating Eureka lemon defence against CYVCV by promoting the ROS and JA pathways. In addition, ClBeclin1 positively regulates Eureka lemon innate immunity, probably by degrading ClAPX1 via the autophagy pathway.

## 2 Results

### 2.1 ClBeclin1 Acts as a Positive Regulator in Eureka Lemon Against CYVCV

**FIGURE 1:**
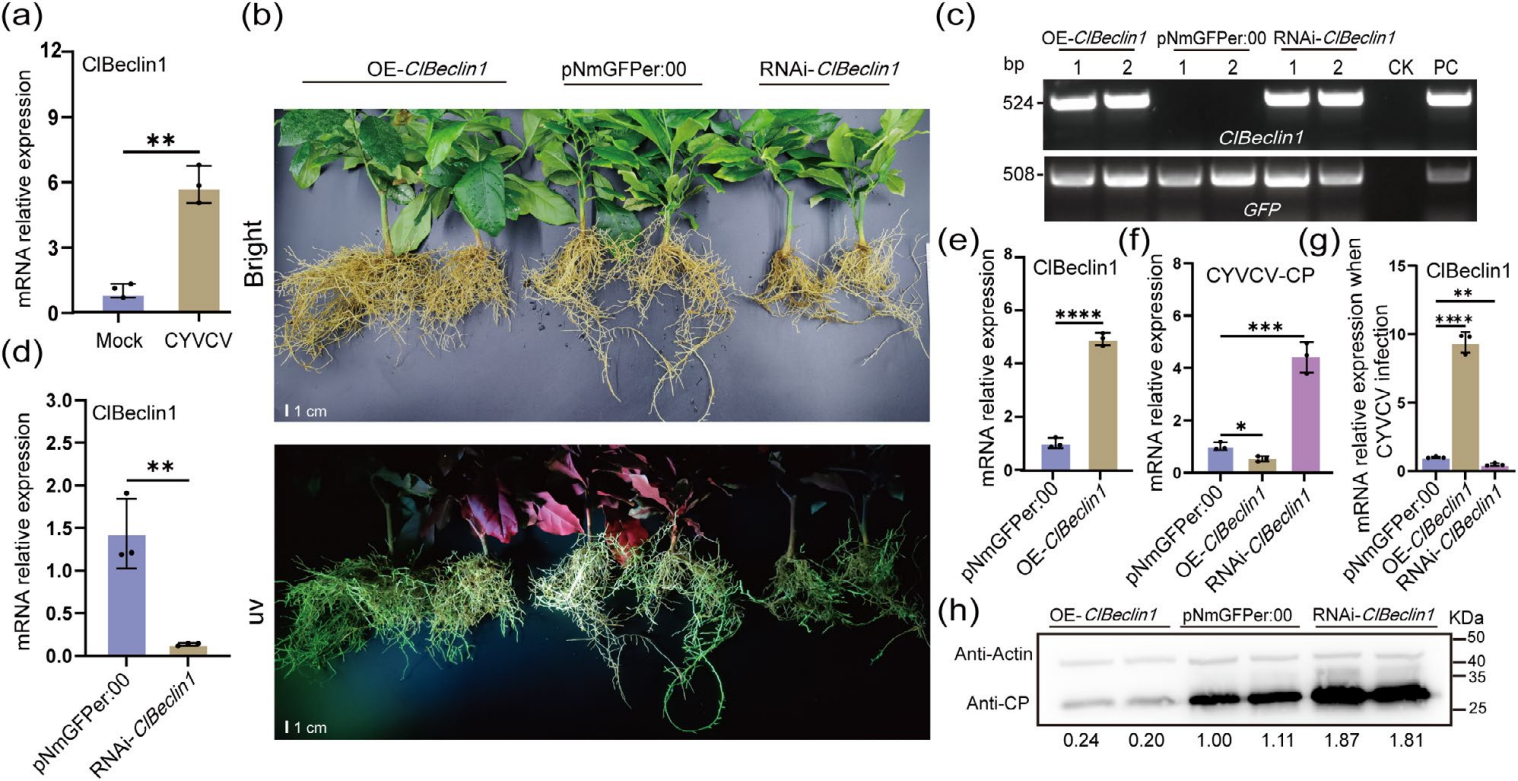
The functional identification of *ClBeclin1* in response to CYVCV infection. (a) The expression pattern of *ClBeclin1* in citrus after infection with CYVCV. The expression of *ClBeclin1* was determined by reverse transcription‐quantitative PCR (RT‐qPCR) after inoculation of 2‐year‐old Eureka lemons with CYVCV at 15 days post‐inoculation (dpi). Eureka lemons treated with phosphate‐buffered saline (PBS) were used as mock‐inoculated control. (b) Green fluorescent signal visualised by UV light in citrus root tissue. (c) PCR‐diagnostic image of *ClBeclin1* and the fluorescent reporter gene *GFP*. (d) and (e) The relative transcript levels of *ClBeclin1* in OE‐*ClBeclin1* and RNAi‐*ClBeclin1* citrus hairy roots. (g) The relative transcript levels of *ClBeclin1* in OE‐*ClBeclin1* and RNAi‐*ClBeclin1* citrus hairy roots at 15 dpi slash‐inoculated with CYVCV. (f) and (h) The RNA or protein level, respectively, of CYVCV accumulation in OE‐*ClBeclin1* and RNAi‐*ClBeclin1* citrus hairy roots at 15 dpi slash‐inoculated with CYVCV. *CsActin* was used as reference gene for RT‐qPCR and western blot assay. Citrus hairy roots transformed with *Agrobacterium rhizogenes* K599 using empty vector (pNmGFPer:00) as negative control, sterile double‐distilled water as control check (CK) and *A. rhizogenes* K599 transformed with target gene plasmid as positive control (PC). Values are means ± *SD* (*n* = 3, biological repeats). Asterisks represent significant differences by Student's *t* test (*p < 0.05, **p < 0.01, ***p < 0.001, ****p < 0.0001).

Previous studies have shown that Beclin1 interacts with and promotes turnip mosaic virus Nlb autophagic degradation to restrict virus infection (Li et al. 2018). To investigate the *ClBeclin1* response to CYVCV infection, the expression of *ClBeclin1* was assayed in Eureka lemon leaves at 15 days post‐inoculation (dpi) with CYVCV by reverse transcription‐quantitative PCR (RT‐qPCR). Compared to the mock‐inoculated plants, *ClBeclin1* was upregulated about 5.9‐fold in CYVCV‐infected plants (Figure 1a). The amino acid similarity and phylogenetic tree analysis showed that *Beclin1* is highly conserved among different citrus cultivars (Figure S1a,b). The expression level of *ClBeclin1* was higher in healthy Eureka lemon roots than in stems and leaves (Figure S1c).

To further elucidate the role of *ClBeclin1* in CYVCV infection, *ClBeclin1* overexpression (OE‐*ClBeclin1*) and *ClBeclin1* RNA interference (RNAi‐*ClBeclin1*) hairy roots were generated using virus‐free Eureka lemon. The successfully transformed hairy roots were verified with the green fluorescent signals, and the presence of the target construct gene *ClBeclin1* and *GFP* by PCR (Figure 1b,c). Compared to the control infiltrated with pNmGFPer:00 vector, the transcript level of *ClBeclin1* was upregulated about 4.9‐fold in OE‐*ClBeclin1* and downregulated 86.4% in RNAi‐*ClBeclin1* hairy roots on average (Figure 1d,e). Then the OE‐*ClBeclin1* and RNAi‐*ClBeclin1* hairy roots were slash‐inoculated with CYVCV. At 15 dpi, compared to the pNmGFPer:00, the transcript level of CYVCV‐CP was significantly increased in RNAi‐*ClBeclin1* hairy roots, whereas it was dramatically reduced in OE‐*ClBeclin1* hairy roots (Figure 1f). *ClBeclin1* was significantly upregulated in OE‐*ClBeclin1* hairy roots compared to pNmGFPer:00 and RNAi‐*ClBeclin1* (Figure 1g). This result was further confirmed by western blot analysis (WB) with antibodies against CYVCV CP (Figure 1h). Therefore, these results indicated that *ClBeclin1* acts as a positive regulator in Eureka lemon against CYVCV.

### 2.2 Transcriptome Profiling of the OE‐ *ClBeclin1* Hairy Roots Implied the Activation of ROS‐ and JA‐Mediated Defence Responses

**FIGURE 2:**
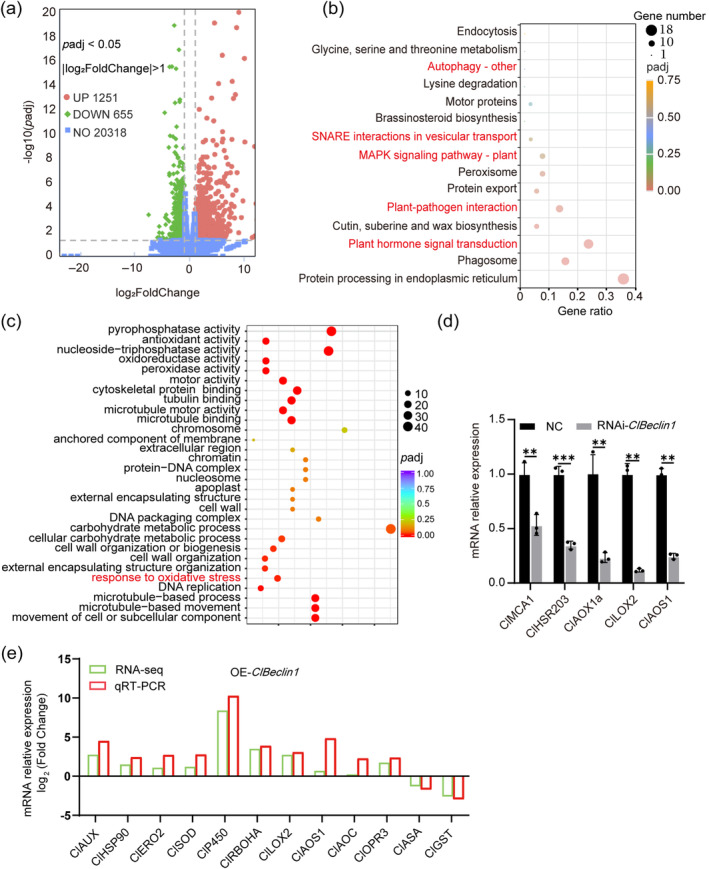
The global transcriptional profiles in OE‐*Beclin1* citrus hairy roots. (a) Volcano plot of differentially expressed genes from pNmGFPer:00 and OE‐*ClBeclin1* citrus hairy roots. (b) Scatter plot of KEGG enrichment. Differentially expressed genes in OE‐*ClBeclin1* and pNmGFPer:00 citrus hairy roots were enriched in 15 KEGG pathways. (c) Scatter plot of Gene Ontology (GO) enrichment analysis. (d) and (e) Validation of transcriptome data using reverse transcription‐quantitative PCR (RT‐qPCR). Random selection of 15 genes involved in the jasmonic acid pathway, oxidative stress and autophagy for RT‐qPCR validation. *CsActin* gene was used as reference for RT‐qPCR. Values are means ± *SD* (*n* = 3, biological repeats). Asterisks represent significant differences by Student's *t* test (**p < 0.01, ***p < 0.001).

To further understand the reprogramming of the virus‐free OE‐*ClBeclin1* hairy roots, RNA‐seq was carried out. Compared to the pNmGFPer:00, 1251 differentially expressed genes (DEGs) were upregulated and 655 DEGs were downregulated in OE‐*ClBeclin1* hairy roots (Figure 2a). KEGG enrichment revealed that 18 DEGs were significantly represented in the process ‘Protein Processing in Endoplasmic Reticulum’, 9 DEGs were significantly represented in the process ‘Plant–Pathogen Interaction’ and 12 DEGs were significantly represented in the process ‘Plant Hormone Signal Transduction’ (Figure 2b). GO enrichment also showed that the biological process ‘Response to oxidative stress’ was significantly upregulated with the overexpression of ClBeclin1 (Figure 2c).

To validate the RNA‐seq results, the expression of 15 DEGs, including ROS‐ (*ClAUX*, *ClHSP90*, *ClERO2*, *ClSOD*, *ClP450*, *ClRBOHA*, *ClMCA1*, *ClAOX1a, ClHSR203, ClASA* and *ClGST*) and JA‐ (*ClLOX2*, *ClAOS1*, *ClAOC* and *ClOPR3*) related genes were examined in OE‐*ClBeclin1* and RNAi‐*ClBeclin1* hairy roots by RT‐qPCR. Among them, ROS positively regulated genes (*ClAUX*, *ClHSP90*, *ClERO2*, *ClSOD*, *ClP450*, *ClRBOHA*, *ClMCA1*, *ClHSR203*) were upregulated, and ROS negatively regulated genes (*ClASA* and *ClGST*) were downregulated; genes involved in JA synthesis (*ClLOX2*, *ClAOS1*) and signalling (*ClAOC*, *ClOPR3*) were upregulated. The expression patterns of these DEGs were consistent with the RNA‐seq results (Figure 2d,e), suggesting the reliability of the RNA‐seq profiling. The changes of these genes indicated that *ClBeclin1* is probably involved in positive regulation of the citrus immunity against CYVCV through ROS‐ and JA‐mediated defence responses.

### 2.3 ClBeclin1 Interacts With the ROS Scavenger ClAPX1

**FIGURE 3:**
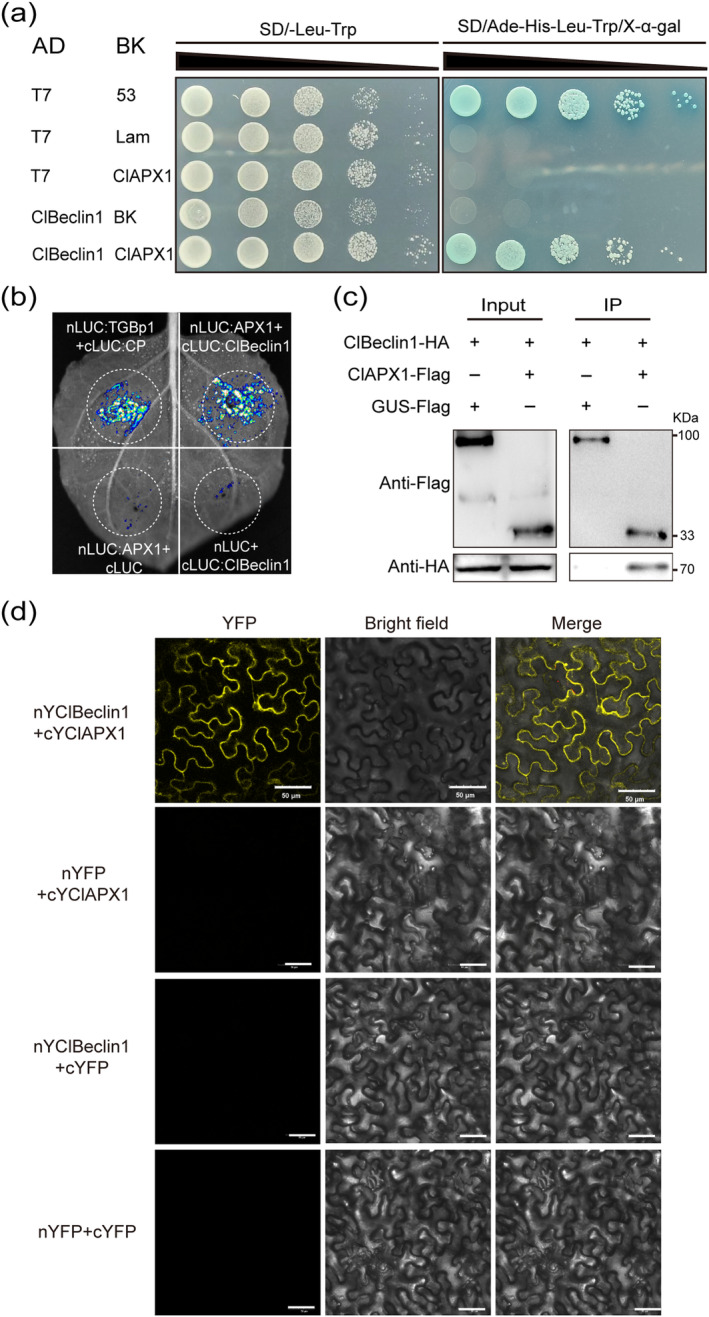
The interaction verification of ClBeclin1 with Eureka lemon ASCORBATE PEROXIDASE1 (APX1). (a) Yeast two‐hybrid assay revealed the interaction between ClBeclin1 and ClAPX1 in yeast cells on SD/−Trp/−Leu and SD/−Trp/−Leu/−His/−Ade/X‐α‐Gal media. BK‐53 and AD‐T were used as positive control and BK‐53 and AD‐Lam as negative control. (b) ClBeclin1 was shown to interact with ClAPX1 in plants using luciferase complementation imaging (LCI) assays. The image shows the luminescence signals detected in *Nicotiana benthamiana* leaves co‐expressing nLUC:ClAPX1 and ClBeclin1:cLUC or the positive control (CP:cLUC + nLUC:TGBp1), while no signal was detected in various negative controls (nLUC:ClAPX1 and cLUC, nLUC and ClBeclin1:cLUC). (c) Co‐immunoprecipitation assay shows that ClBeclin1 and ClAPX1 interact. Total protein extracts were immunoprecipitated with anti‐FLAG beads. This was followed by immunoblotting with anti‐FLAG or anti‐HA antibodies. (d) The interaction between ClBeclin1 and ClAPX1 was demonstrated in a bimolecular fluorescence complementation assay. The yellow fluorescent protein (YFP) signal was visualised by confocal microscopy in nYFP:ClBeclin1 + ClAPX1:cYFP, but not in the negative control (nYFP+ClAPX1:cYFP, cYFP+nYFP:ClBeclin1, nYFP+cYFP). Scale bar = 50 μm. The experiments were repeated three times with similar results.

Previous studies have suggested that Beclin1 can interact with necrosis inhibitors to regulate plant immunity (Xu et al. 2017), while APX, similar to death suppressor factors, may be involved in plant immunity by regulating ROS and JA (Zhang et al. 2022; Wang et al. 2023). Therefore, we speculated that the inhibition of CYVCV infection mediated by ClBeclin1 might be facilitated through its interaction with ClAPXs. To examine this hypothesis, yeast strains carrying the pGBKT7:ClAPX1‐7 and pGADT7:ClBeclin1 vectors were grown on selective media (SD/−Leu/−Trp and SD/−Leu/−Trp/−His/−Ade with 20 μg/mL X‐α‐Gal). The result suggests the interaction of ClBeclin1 and ClAPX1 in the yeast two‐hybrid (Y2H) assay (Figure 3a and Figure S2a). Their interaction was then confirmed by a luciferase complementation imaging (LCI) assay. ClBeclin1 fused with C‐terminal luciferase (ClBeclin1:cLUC) was transiently co‐expressed with ClAPX1 fused with the N‐terminal luciferase (nLUC:ClAPX1) in *Nicotiana benthamiana*. Strong luciferase (LUC) activity was detected in the combination of nLUC:ClAPX1 and cLUC:ClBeclin1, as well as in the positive control (cLUC:CP and nLUC:TGBp1) at 48 h post‐infection (hpi) (Figure 3b). Furthermore, their interaction was confirmed with a bimolecular fluorescence complementation (BiFC) assay. Compared to nYFP + cYFP:ClAPX1, nYFP:ClBeclin + cYFP and nYFP + cYFP controls, yellow fluorescent signal was only observed in the cytoplasm of *N. benthamiana* after co‐infiltration of cYFP:ClAPX1 and nYFP:ClBeclin (Figure 3d). Moreover, a co‐immunoprecipitation (Co‐IP) assay was performed. The 3HA‐tagged ClBeclin1 (3HA:ClBeclin1) was co‐immunoprecipitated with 3 × FLAG‐tagged ClAPX1 (ClAPX1:3Flag), but not with 3 × FLAG‐tagged β‐glucuronidase (GUS) (Figure 3c). Thus, ClBeclin1 interacts with the ROS scavenger ClAPX1.

To identify the critical interaction domain of ClBeclin1 with ClAPX1, ClBeclin1 was truncated into three fragments (ClBeclin1‐N^1‐167^, ClBeclin1‐CC^168‐296^ and ClBeclin1‐BARA^297‐515^) on the basis of its conserved domains (Figure S2b). Y2H assay showed that ClBeclin1‐CC^168‐296^ interacted with ClAPX1 (Figure S2c). BiFC further confirmed that ClBeclin1‐CC^168‐296^ and ClAPX1 interacted in *N. benthamiana* (Figure S2d).

### 2.4 Subcellular Location of ClBeclin1 and ClAPX1

**FIGURE 4:**
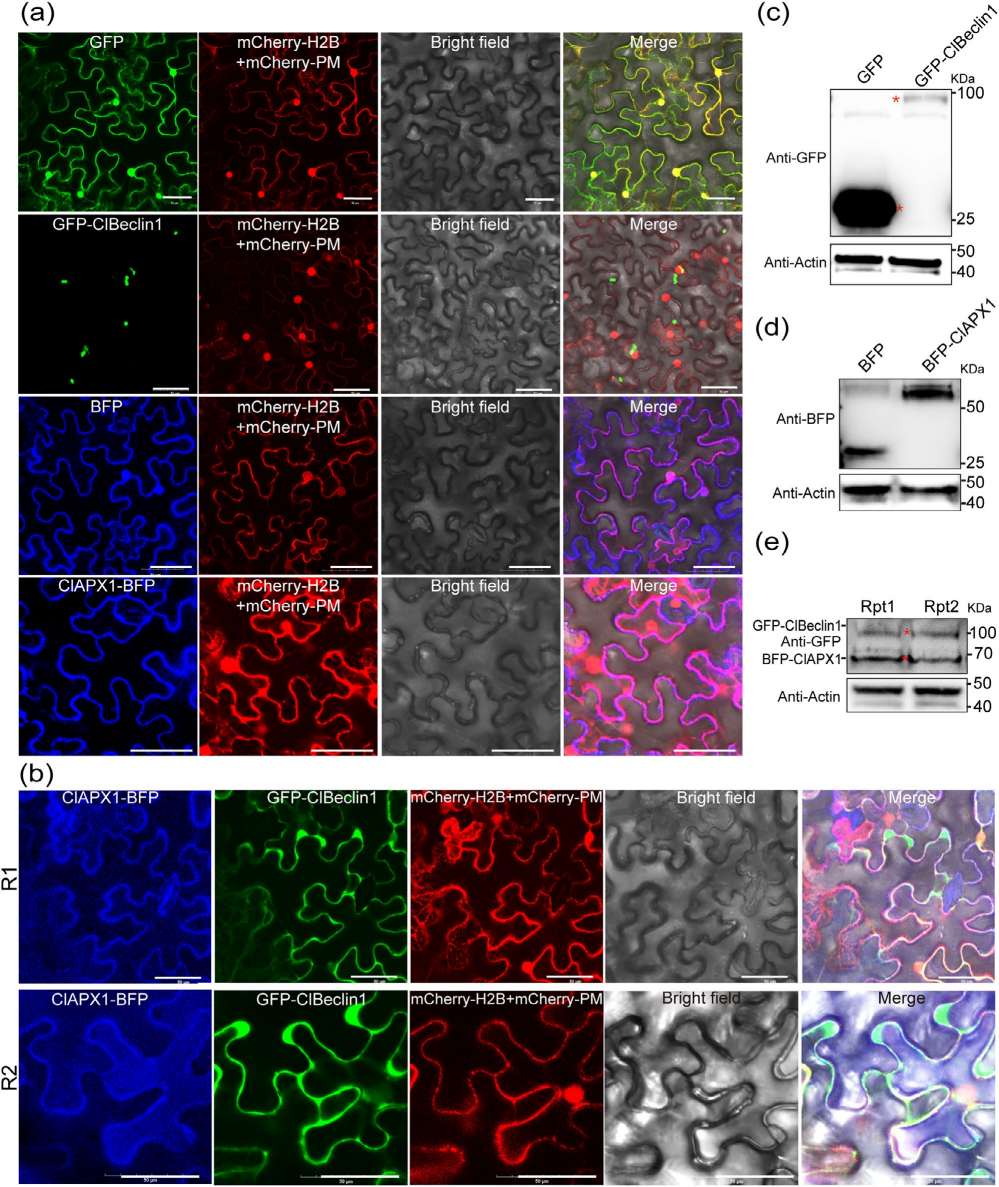
Analysis of the subcellular localisation of ClBeclin1 and ClAPX1 in *Nicotiana benthamiana*. (a) ClBeclin1 is localised in punctate structures within the cytoplasm and ClaAPX1 is localised at the cytoplasm. Both GFP and BFP empty vectors control were localised in the nucleus and cytoplasm. (b) Co‐localisation of ClBeclin1 with ClAPX1 at the cytoplasm. (c–e) Protein extraction was performed for western blot analysis to detect the expression of subcellular localisation proteins in *N. benthamiana* leaves. The expression of GFP and BFP was confirmed by the use of anti‐GFP antibodies; CsActin was used as a reference loading control. mCherry‐H2B and mCherry‐PM (CD3‐1007‐RFP) were used as nuclear and plasma membrane indicators, respectively. Scale bar = 50 μm. The experiment included at least three technical replicates and at least 10 fields of view per plant were observed with similar results.

To reveal the subcellular localisation of ClBeclin1 and ClAPX1, GFP:ClBeclin1 or ClAPX1:BFP were separately expressed with the nuclear marker H2B‐mCherry and the plasma membrane marker CD3‐1007‐RFP in *N. benthamiana* leaves. At 48 hpi, confocal microscopy observation showed that GFP:ClBeclin1 was localised in a punctate structure in the cytoplasm, while ClAPX1:BFP was localised in the cytoplasm and appeared in a dispersed state (Figure 4a). When GFP:ClBeclin1 and ClAPX1:BFP were co‐expressed, they co‐localised in the cytoplasm in a dispersed state (Figure 4b), consistent with BiFC observations (Figure 3d). The correct expression of the target proteins was confirmed by WB assay (Figure 4c–e).

Because the CC^168‐296^ domain is the key region interacting with ClAPX1, its subcellular localisation was also determined in the cytoplasm (Figure S3a). To further verify the subcellular localisation of CC^168‐296^:eGFP, the cytoplasmic and cytoplasmic membrane fraction proteins were extracted separately for WB assay. The results showed that both CC^168‐296^:eGFP and GFP were detected only in the cytoplasmic fraction (Figure S3b). Collectively, ClBeclin1 colocalises with ClAPX1 in the cytoplasm through the CC^168‐296^ domain.

### 2.5 ClBeclin1 Inhibits the Enzymatic Activity of ClAPX1 Through Promoting Its Autophagic Degradation

**FIGURE 5:**
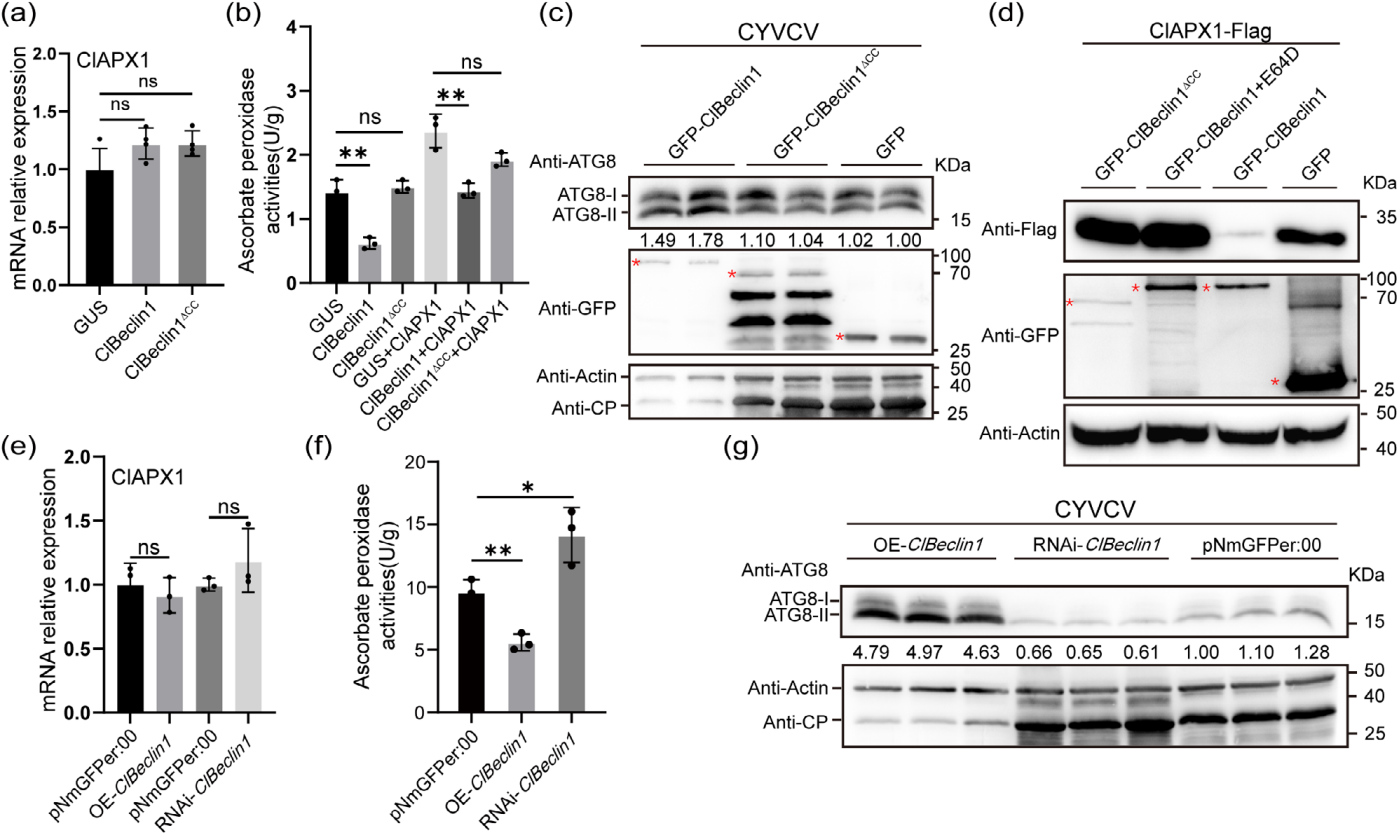
ClBeclin1 mediates the autophagic degradation of ClAPX1 and inhibits its enzymatic activity. (a) and (b) The effect of ClBeclin1 on the transcription level and enzymatic activity of ClAPX1. (c) The effect of ClBeclin1 on autophagy and CYVCV coat protein (CP) levels. (d) ClBeclin1 targets ClAPX1 for autophagic degradation. eGFP/eGFP:ClBeclin1/eGFP:ClBeclin1^∆CC^ with ClAPX1:FLAG were co‐infiltrated into Eureka lemon leaves by *Agrobacterium*‐mediated transformation. The autophagy inhibitor E64D was infiltrated into Eureka lemon leaves co‐expressing eGFP:ClBeclin1+ ClAPX1:FLAG after 12 h. (e–g) Expression and enzymatic activity of ClAPX1, CP and autophagy levels in OE‐*ClBeclin1*, RNAi‐*ClBeclin1* and pNmGFPer:00 citrus hairy roots. Anti‐GFP, anti‐FLAG, anti‐ATG8 and anti‐CsActin antibodies were used in the western blot assay. *CsActin* gene was used as reference for reverse transcription‐quantitative PCR. Asterisks represent significant differences by Student's *t* test (*p < 0.05, **p < 0.01, ns, not significant). Values are means ± *SD* (*n* = 3 technical repeats). All experiments were repeated three times independently.

To determine how ClBeclin1 affects ClAPX1, the transcript level of *ClAPX1* was first determined by RT‐qPCR when eGFP:ClBeclin1, eGFP:ClBeclin1^∆CC^ or eGFP was expressed in Eureka lemon leaves at 15 dpi with CYVCV infection. The result showed that ClBeclin1 and ClBeclin1^∆CC^ did not alter the expression of *ClAPX1* compared to co‐expression with eGFP (Figure 5a). While the enzymatic activity of ClAPX1 was significantly reduced when eGFP:ClBeclin1 was co‐expressed with ClAPX1:FLAG or alone, no change was observed when eGFP:ClBeclin1^∆CC^ was co‐expressed with ClAPX1:FLAG or alone compared to the controls (GUS or ClAPX1:FLAG+GUS; Figure 5b). APX has seven homologous proteins (ClAPX1–ClAPX7) in lemon (Zhang, Song, et al. 2023). To further clarify whether the effect of ClBeclin1 on enzyme activity is specific to ClAPX1, ClAPX1–ClAPX7:FLAG were transiently co‐expressed with ClBeclin1:HA or empty vector (EV) in Eureka lemon leaves. As shown in Figure S4c,d, only the enzyme activity of ClAPX1 was significantly reduced by ClBeclin1 compared to the control. These results indicated that ClBeclin1 can significantly reduce the enzyme activity of ClAPX1 instead of other ClAPX members.

Considering that Beclin1 is involved in the autophagic degradation pathway, we tested whether ClBeclin1 affects the enzymatic activity of ClAPX1 by degrading it via autophagy. *ClBeclin1*, *ClBeclin1* ^∆CC^ and *GFP* were transiently expressed in Eureka lemon infected with CYVCV. At 48 dpi, the protein levels of the autophagy marker ATG8‐II and CP were significantly increased and decreased in Eureka lemon leaves overexpressing *ClBeclin1* compared to Eureka lemon leaves overexpressing *GFP* or *ClBeclin1* ^*∆*CC^, respectively (Figure 5c). The result robustly demonstrates that *ClBeclin1* promotes autophagy activity and limits CYVCV infection in Eureka lemon plants. Subsequently, ClAPX1:FLAG was co‐expressed with eGFP, eGFP:ClBeclin1, eGFP:ClBeclin1 + E64D or eGFP:ClBeclin1^∆CC^ in Eureka lemon leaves, and the protein level was assayed with WB. Compared to the controls (ClAPX1:FLAG + eGFP), the abundance of ClAPX1:FLAG was remarkably decreased when it was co‐expressed with eGFP:ClBeclin1, while the decrease of ClAPX1:FLAG was inhibited by autophagy inhibitor E64D treatment (Figure 5d). However, degradation of ClAPX1:FLAG was not observed when it was co‐expressed with eGFP:ClBeclin1^∆CC^ (Figure 5d). The CYVCV CP protein can interact with ClAPX1 to promote viral infection (Wang et al. 2023). To clarify whether autophagic degradation of ClAPX1 by ClBeclin1 affects the CP level, ClBeclin1:HA and CP were co‐expressed in the presence or absence of ClAPX1:FLAG under the conditions of E64D or dimethyl sulphoxide (DMSO) treatment in Eureka lemon leaves. It was found that ClBeclin1–ClAPX1 interaction significantly promoted the degradation of CP (Figure S4a), whereas CP degradation did not occur with ClAPX1 alone (Figure S4b). These results suggest that ClBeclin1 promoted the autophagic degradation of ClAPX1 via the CC^168‐296^ domain, and that the degradation of ClAPX1 further affects the content of CYVCV CP.

In addition, the mRNA levels and enzymatic activity of ClAPX1 were examined in OE‐*ClBeclin1* and RNAi‐*ClBeclin1* hairy roots. As expected, the transcriptional level of *ClAPX1* did not change (Figure 5e), while its enzymatic activity was significantly inhibited in OE‐*ClBeclin1*, but substantially increased in RNAi‐*ClBeclin1* hairy roots compared to the pNmGFPer:00 (Figure 5f). Furthermore, the abundance of ATG8‐II protein was significantly increased in OE‐*ClBeclin1*, whereas it was significantly lower than in RNAi‐*ClBeclin1* compared to the pNmGFPer:00, and the accumulation level of CP showed the opposite pattern (Figure 5g). Taken together, our results suggest that ClBeclin1 affects its enzymatic activity through autophagic degradation of ClAPX1 and that the CC^168‐296^ domain of ClBeclin1 is involved in the degradation of ClAPX1. ClBeclin1 can promote the autophagic degradation of ClAPX1, which in turn facilitates the degradation of the CYVCV CP.

### 2.6 ClBeclin1 Inhibits CYVCV Replication by Activation of ROS‐Mediated Basal Defence Responses

**FIGURE 6:**
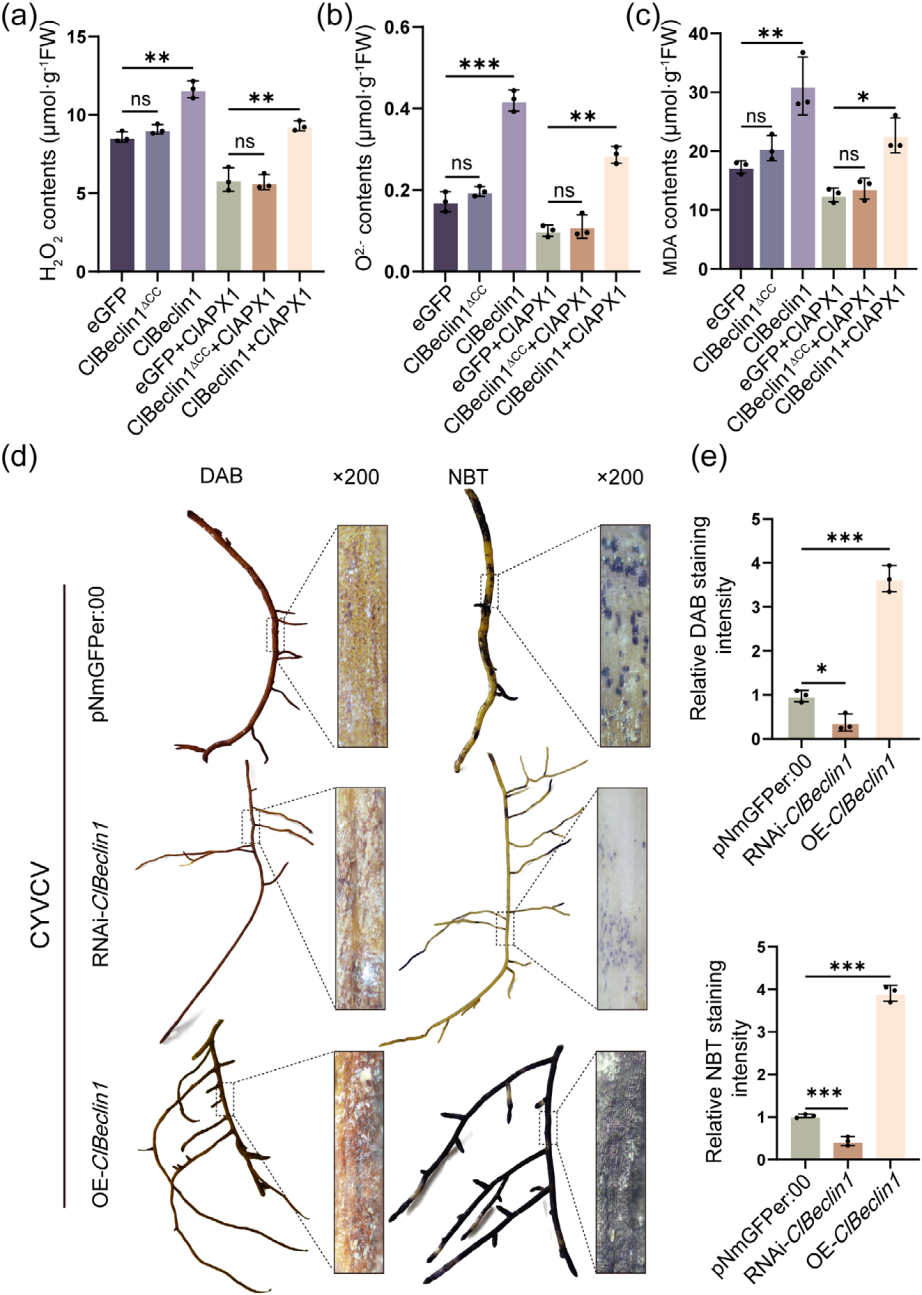
Overexpression of *ClBeclin1* can induce an oxidative stress response. (a–c) Overexpression of *ClBeclin1* could induce the levels of H_2_O_2_, O_2_^·−^ and malondialdehyde (MDA). eGFP/eGFP:ClBeclin1/eGFP:ClBeclin1^∆CC^ infiltrated into Eureka lemon leaves alone or with ClAPX1:FLAG by *Agrobacterium*‐mediated transformation. Detection of reactive oxygen species (ROS) levels after 48 h. (d) and (e) ROS production in OE‐*ClBeclin1*, RNAi‐*ClBeclin1* and pNmGFPer:00 citrus hairy roots infected with CYVCV was analysed by 3,3′‐diaminobenzidine (DAB) and nitroblue tetrazolium (NBT) staining. Values were obtained by measuring three representative images selected from three biological replicates, and data presented are means ± *SD*. Statistical analysis was performed using Student's *t* test (*p < 0.05, **p < 0.01, ***p < 0.001).

The function of APX is to remove H_2_O_2_ from plant tissue (Du et al. 2023). To investigate whether the autophagic degradation of ClAPX1 by ClBeclin1 affects ROS levels, the levels of H_2_O_2_, O_2_^−^ and malondialdehyde (MDA) were determined after eGFP:ClBeclin1, eGFP:ClBeclin1^∆CC^ or eGFP were co‐expressed with ClAPX1:FLAG or alone in Eureka lemon leaves at 15 dpi with CYVCV infection. The results showed that the levels of H_2_O_2_, O_2_^−^ and MDA were significantly increased in Eureka lemon leaves when eGFP:ClBeclin1 was co‐expressed with ClAPX1:FLAG or alone, whereas the levels were not changed when eGFP:ClBeclin1^∆CC^ was co‐expressed with ClAPX1:FLAG or alone compared to the controls (eGFP or ClAPX1:FLAG + eGFP) (Figure 6a–c). The ROS levels were also measured in OE‐*ClBeclin1* and RNAi‐*ClBeclin1* hairy roots at 15 dpi with CYVCV infection by 3,3'‐diaminobenzidine (DAB) and nitroblue tetrazolium (NBT) staining. Compared to the pNmGFPer:00, ROS accumulated at a significantly higher level in OE‐*ClBeclin1* and at a markedly lower level in RNAi‐*ClBeclin1* hairy roots (Figure 6d,e). These results indicate that the overexpression of *ClBeclin1* promotes the ROS content accumulation through inhibition of ClAPX1 enzyme activity in Eureka lemon.

To further clarify whether ROS accumulation confers resistance to CYVCV, exogenous H_2_O_2_ was sprayed on CYVCV‐infected Eureka lemon young leaves. As high concentrations of H_2_O_2_ can be detrimental to plant leaves (Wu et al. 2020), five different H_2_O_2_ concentrations (5, 10, 50, 100 and 500 mM) were tested. After 15 days of spraying 100 and 500 mM H_2_O_2_, necrosis symptoms were observed on the treated Eureka lemon leaves (Figure S4a). Therefore, 50 mM H_2_O_2_ was applied to CYVCV‐infected Eureka lemons. At 15 dpi, Eureka lemons treated with 50 mM H_2_O_2_ showed mild vein clearing symptoms compared to controls (Figure S4b). WB and RT‐qPCR assay also showed that protein and RNA levels of CYVCV CP were significantly lower than in control Eureka lemons (Figure S4c,d). In conclusion, the results indicated that the overexpression of *ClBeclin1* can inhibit CYVCV infection by activating the ROS‐mediated basal defence response.

### 2.7 ClBeclin1 Inhibits CYVCV Replication Through Activation of JA‐Mediated Defence Responses

**FIGURE 7:**
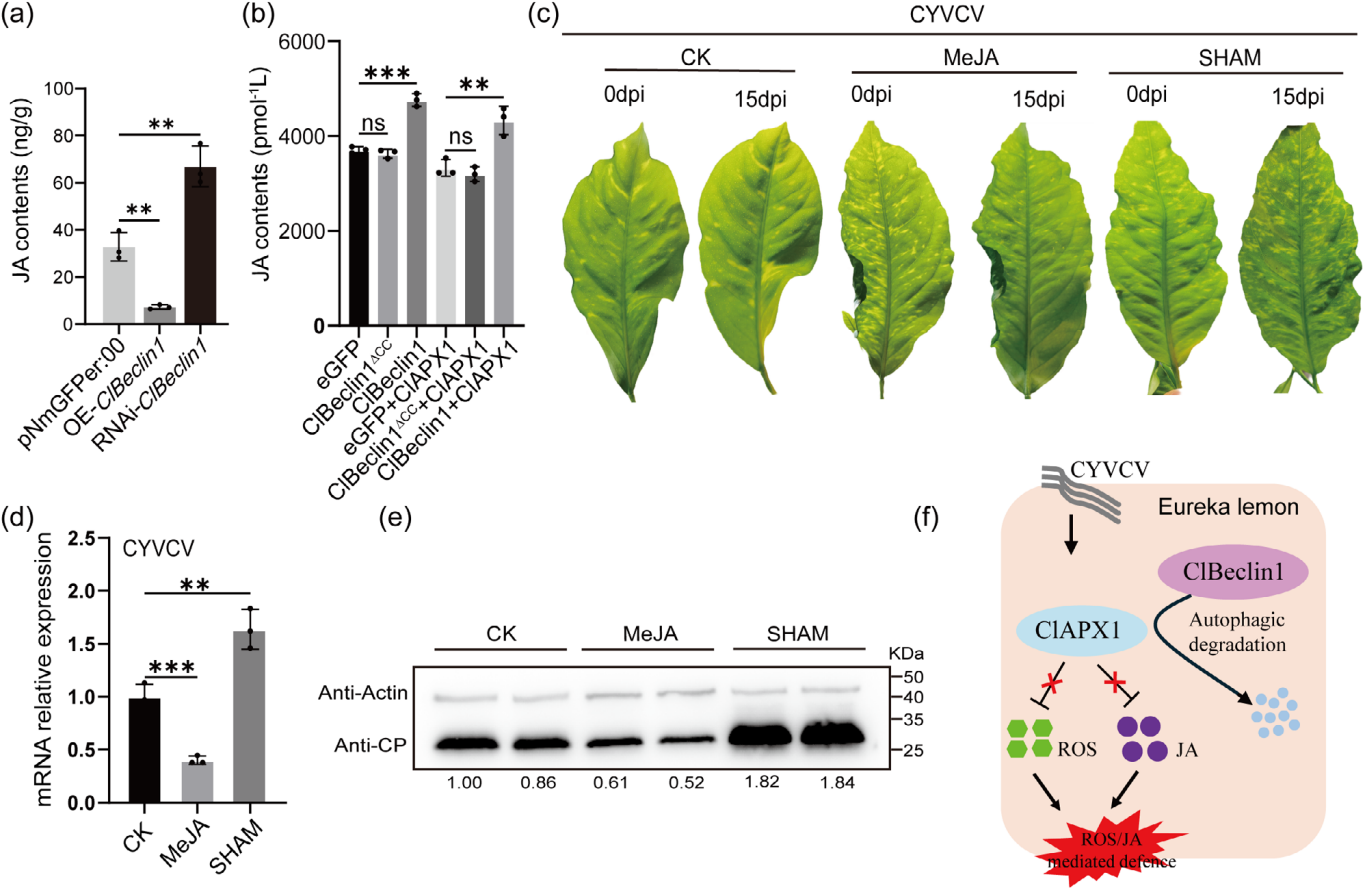
Overexpression of *ClBeclin1* activates the jasmonic acid (JA) pathway and confers antiviral activity. (a) JA accumulation in OE‐*ClBeclin1*, RNAi‐*ClBeclin1* and pNmGFPer:00 citrus hairy roots. (b) Effect of ClBeclin1 on JA levels in Eureka lemon. (c) Phenotypic symptoms following treatment of CYVCV‐infected Eureka lemon leaves with ethanol‐water (CK), 100 μM methyl jasmonate (MeJA) and 100 μM salicylhydroxamic acid (SHAM). Treatments are given every 3 days for 15 days. The photographs were taken at 15 days post‐inoculation. (d) and (e) The RNA or protein level of CYVCV accumulation in citrus plants after treatment with ethanol‐water (CK), MeJA and SHAM. (f) A working model of the ClBeclin1‐mediated degradation of ClAPX1 in Eureka lemon immunity. Values are means ± *SD* (*n* = 3 technical repeats). Asterisks represent significant differences by Student's *t* test (*p < 0.05, **p < 0.01, ***p < 0.001). Anti‐coat protein (CP) and anti‐CsActin antibodies were used in the western blot assay.

Wang et al. (2023) found that *ClAPX1* acts as a positive regulator against CYVCV infection in Eureka lemon by inhibiting the expression of JA‐mediated genes. *ClBeclin1* was identified as a positive regulator against CYVCV infection and activated the expression of JA synthesis genes in OE‐*ClBeclin1* citrus hairy roots (Figures 1g,h and 2e). Subsequently, JA content was measured in citrus hairy roots. Compared to the pNmGFPer:00, the JA content was increased 2.0‐fold in OE‐*ClBeclin1*, but decreased 4.5‐fold in RNAi‐*ClBeclin1* hairy roots instead (Figure 7a). To clarify whether the increase in JA levels with *ClBeclin1* overexpression was caused by autophagic degradation of ClAPX1, JA content was determined in citrus leaves when eGFP:ClBeclin1, eGFP:ClBeclin1^∆CC^ or eGFP was co‐expressed with ClAPX1:FLAG or alone in Eureka lemon leaves at 15 dpi with CYVCV infection. The JA content was significantly increased in Eureka lemon leaves when eGFP:ClBeclin1 was co‐expressed with ClAPX1:FLAG or alone, whereas the JA levels were not changed when eGFP:ClBeclin1^∆CC^ was co‐expressed with ClAPX1:FLAG or alone compared to the controls (eGFP or ClAPX1:FLAG+eGFP) (Figure 7b).

To further clarify the role of JA in mediating resistance to CYVCV, CYVCV‐infected plants were treated with exogenous 100 μΜ methyl jasmonate (MeJA, a derivative of JA) or salicylhydroxamic acid (SHAM, JA synthesis inhibitor) (Figure 7c). Compared to the controls (treated with ethanol‐water), the mRNA and protein levels of CYVCV CP were significantly reduced in MeJA‐treated leaves, whereas they were remarkably increased in SHAM‐treated leaves (Figure 7d,e). Therefore, these results suggested that ClBeclin1 may activate JA‐mediated defence responses by autophagic degradation of ClAPX1, thereby inhibiting CYVCV replication.

## 3 Discussion

*Beclin1*, a key gene involved in the process of autophagy, can bind to certain viral proteins and restrict viral infection through the autophagy pathway (Yue et al. 2015; Li et al. 2018; Huang et al. 2024). *Beclin1* can also negatively regulate *N*‐gene‐mediated programmed cell death (Liu et al. 2005; Patel and Dinesh‐Kumar 2008), while the regulation of ROS by *Beclin1* in compatible plant–virus interactions remains largely unexplored. In this study, overexpression of *ClBeclin1* promoted the accumulation of ROS induced by CYVCV. In contrast, we found that *Beclin1* acts as a positive regulator of ROS induced by compatible plant viruses, and suggested that the regulation of ROS by *Beclin1* may be different in incompatible and compatible plant pathogen defence responses. Our findings help to highlight the functional diversity of the *Beclin1* in regulating ROS in plants and improve our understanding of the involvement of the *Beclin1* in plant defence responses.

ROS are some of the earliest activated signalling molecules and generally play positive roles against viral infection. Plants can promote ROS accumulation under biotic stress by regulating ROS scavenging mechanisms. For example, wheat TaVTC2 reduces its own enzymatic activity by recruiting wheat yellow mosaic virus (WYMV) NIb protein, leading to ascorbic acid reduction and thereby evoking a ROS burst, which confers resistance to WYMV infection (Zhang, Hu, et al. 2023). During Chinese wheat mosaic virus infection, the TaTHI2 protein accumulates to facilitate ROS production by suppressing TaCPK5‐mediated activity of the catalase protein TaCAT1, limiting viral infection (Yang et al. 2024). In the current study, ClBeclin1 was found to reduce the enzymatic activity of ClAPX1 by affecting its stability through autophagic degradation. Moreover, the CC^168‐296^ domain, which encodes the resistance (*R*) gene of NLRs in induction of cell death and enhancement of resistance to viruses (Horsefield et al. 2019; Baudin et al. 2020; Wu et al. 2023), is tightly associated with ClBeclin1 function in suppression of the enzymatic activity of ClAPX1. However, the enzymatic activity of ClAPX1 was demonstrated to be enhanced due to its recruitment by CYVCV CP, resulting in enhancement of ROS scavenging and thereby assisting CYVCV infection (Wang et al. 2023). ClBeclin1 was found to restrict CYVCV infection via ClAPX1 as a bridge. *Beclin1* defence against pathogens occurs mainly in the early stages of pathogen invasion (Patel and Dinesh‐Kumar 2008). Similarly, OE‐*ClBeclin1* was found to delay the time of CYVCV entry but does not seem to have an effect in the later stages of infection. This may be because the virus successfully enters and produces a large amount of CP and ClBeclin1 to bind competitively to ClAPX1, thereby promoting infection. In summary, the plant–virus interaction is a fierce molecular arms race (Wu et al. 2024) in which ROS‐based basal defence responses highly contribute to the ultimate outcome of CYVCV infection and replication.

In addition to ROS, plant hormones are also vital players in plant immunity (Wang et al. 2021). For example, the accumulation of JA enhances plant defence against rice stripe virus (Han et al. 2020). Similarly, exogenous application of JA enhances plant resistance to viruses (Zhu et al. 2014; He et al. 2017; Tan et al. 2022). Meanwhile, increasing evidence suggests that JA regulates antioxidant metabolism. High levels of JA induce the overaccumulation of ROS and suppress of the activities of antioxidant enzymes (Yu et al. 2022). JA modulates ROS homeostasis to restrict *Rhizoctonia solani* AG1‐IA infection in rice (Kumar et al. 2023). The JA signalling pathway mediated by CsJAZ1‐CsMYC2.2 modulates tea plant susceptibility to *Colletotrichum camelliae* by regulating *CsGSTU45* to accumulate H_2_O_2_ (Lv et al. 2024). These studies implied the cross‐talk between JA and ROS signalling pathways during plant–pathogen interactions (Huang et al. 2019; Devireddy et al. 2021). Of particular interest, recently it has been found that ROS can stimulate biosynthesis of JA as well (Geng et al. 2024). Maize ZmAPX1 confers resistance to Southern corn leaf blight by decreasing H_2_O_2_ accumulation and activating the JA‐mediated defence signalling pathway (Zhang et al. 2022). ClAPX1 negatively regulates genes involved in the JA‐mediated signalling pathways (Wang et al. 2023). However, the mechanism by which the APX modulates JA‐mediated plant immunity against pathogens needs further exploration.

Meanwhile, other possibilities are not excluded. In the autophagy process, Beclin1 acts as a selective autophagy receptor and interacts with ATG8a to participate in the degradation of autophagic substrates (Li et al. 2018; Huang et al. 2024). Recently, a study has demonstrated that a negative regulator of JA signalling, JASMONATE‐ASSOCIATED MYC2‐LIKE 1 (JAM1), is degraded through the autophagy pathway by interacting with ATG8a, which activates the JA signalling pathway and enhances resistance to *Meloidogyne incognita* (Zou et al. 2023). This implies that ClBeclin1 may not need to target the autophagic degradation of ClAPX1 to participate in the regulation of ROS and JA, but can instead target the autophagic degradation of the negative regulator of JA using ATG8a as a bridge to participate in the regulation of JA or ROS levels. Nevertheless, whether regulators in JA signalling are targeted for autophagic degradation requires further investigation.

In summary, ROS and JA are important players in plant immunity signalling and they function against CYVCV infection. A working model is proposed in Figure 7f. ClBeclin1 interacts with ClAPX1 and promotes its degradation via the autophagy pathway, resulting in the activation of both ROS‐ and JA‐mediated defence responses, leading to enhanced resistance to CYVCV.

## 4 Experimental Procedures

### 4.1 Plant Materials and Growth Conditions

*Nicotiana benthamiana* plants were grown under conditions of 65% ± 2% relative humidity, 25°C ± 2°C, 16 h of light and 8 h of darkness. Healthy and CYVCV‐infected Eureka lemons and their hairy root plants were grown in a greenhouse with a 16‐h light/8‐h dark cycle and 60% relative humidity.

### 4.2 Bioinformatics Analysis of ClBeclin1

Amino acid sequences of different citrus varieties of Beclin1 were retrieved from https://www.citrusgenomedb.org/ and http://citrus.hzau.edu.cn/ database. The conserved domains of ClBeclin1 were predicted with SMART (http://smart.embl‐Heidelberg.de/) and InterPro (https://www.ebi.ac.uk/interpro/). Phylogenetic analysis of Beclin1 was conducted with Sequence Demarcation Tool v. 1.3, and a phylogeny tree was constructed by MEGA X.

### 4.3 Eureka Lemon Hairy Root Transformation

For overexpression of *ClBeclin1*, primer pairs OE‐*ClBeclin1*‐F and OE‐*ClBeclin1*‐R were used to amplify the coding sequence of *ClBeclin1* using the PrimeSTAR high‐fidelity enzyme (TaKaRa) and then cloned into the KpnI‐ and SalI‐digested pNmGFPer vector to generate the pNmGFPer:ClBeclin1 expression vector. Construction of the ClBeclin1 interference vector (*ClBeclin*1‐RNAi) was performed according to the protocol described in Zou et al. (2021). The constructs were introduced into Eureka lemon plants by *Agrobacterium rhizogenes* K599 (pSoup)‐mediated transformation as described (Ramasamy et al. 2023; Xiao et al. 2023). Identification of the gene transformation and subsequent experiments were carried out at approximately 120 dpi. All primers used for vector construction in this study are listed in Table S1.

### 4.4 PCR and RT‐qPCR

Total DNA was extracted by Biospin Omni Plant Genomic DNA Extraction Kit (BioFlux), and the Tanon 5200 multi fully automated imaging system was used for PCR imaging. Total RNA was extracted by RNAiso Plus (TaKaRa) and was reverse transcribed into cDNA by the All‐In‐One 5× RT MasterMix (ABM). qPCR was performed using a q225 real‐time PCR machine (Novogene) and a SYBR Prime qPCR Set (Bioground). The 2^−ΔΔCT^ method was used to calculate the relative expression levels of the target genes (Livak and Schmittgen 2001), and *CsActin* was used as the internal control (Li et al. 2020). The statistical analysis and plotting of the data were performed using GraphPad Prism v. 9.0 software (GraphPad Software Inc.). All primers used were listed in Table S1.

### 4.5 Western Blot Assay

Leaf tissues were homogenised in lysis buffer (Solarbio) to extract total proteins. Protein samples were then mixed with 5× SDS loading buffer and boiled for 10 min. Then protein samples were separated with 12.5% SDS‐PAGE, and ATG8 protein was separated with 15% SDS‐PAGE on 6 M urea gels and transferred onto polyvinylidene fluoride (PVDF) membranes (Yoshimoto et al. 2004). Antibodies against CP (Genecrate), FLAG (Proteintech), HA (Proteintech), GFP (Alpvhh), actin (Abbkin) and ATG8 (Agrisera) were used for blot detection, followed by incubation with a horseradish peroxidase‐conjugated anti‐mouse/anti‐rabbit secondary antibody.

### 4.6 Yeast Two‐Hybrid Assay

Y2H was performed according to the manufacturer's instructions (https://www.weidibio.com/upload/file/20230615/20230615201927_24543.pdf). Specifically, the constructed plasmids pGADT7:ClBeclin1, pGBKT7:ClAPX_1‐7_, pGADT7:N, pGADT7: CC^168‐296^ and pGADT7:BARA were transformed into *Saccharomyces cerevisiae* AH109 competent cells, and then 10 μL carrier DNA was added with 500 μL polyethylene glycol/lithium acetate and mixed by aspiration several times. The strains were cultured on SD (−Trp/−Leu) and SD (−Trp−Leu−His−Ade with 40 μg/mL X‐α‐gal) agar plates at 29°C for 3–7 days to verify the interaction. Yeast cells transformed with AD‐T and BD‐Lam were used as negative controls, while yeast cells transformed with AD‐T and BD‐53 were used as positive controls.

### 4.7 Bimolecular Fluorescence Complementation Assay

The constructed plasmids pCV‐cYFP‐ClAPX1 and pCV‐nYFP‐ClBeclin1 were separately transformed into *Agrobacterium tumefaciens* GV3101 (pSoup). *Agrobacterium* cultures were centrifuged, resuspended in inoculation buffer (OD_600_ = 1.0), mixed in equal volumes and incubated for 2 h in the dark and then infiltrated into *N. benthamiana* leaves. The inoculated leaves were collected with a 5 mm diameter punch and observed under an Olympus FV3000 confocal microscope after 48 h. The excitation wavelength for YFP was 488 nm.

### 4.8 Luciferase Complementation Imaging Assay

The plasmids pCV‐nlUC‐ClAPX1 and pCV‐cLUC‐ClBeclin1 were separately transformed into *A. tumefaciens* GV3101 (pSoup). After cultivation, the concentration was adjusted to OD_600_ = 1.0 using inoculation buffer, incubated for 2 h at room temperature in the dark, and then inoculated into *N. benthamiana* by mixing in equal proportions. Infected leaves were detached after 48 h and the backs of the leaves were sprayed with 1 mM D‐luciferin (MCE) reaction solution. The leaves were incubated in the dark for 7 min and then observed under a chemiluminescence imaging system (Tanon 4600).

### 4.9 Co‐Immunoprecipitation Assay

The *Agrobacterium* cultures containing the citrus transient expression vectors HA:ClBeclin1 and ClAPX1:Flag or GUS:FLAG were infiltrated into Eureka lemon leaves. After 48 h, total protein was extracted from the infiltrated Eureka lemon leaves (Solarbio) and incubated with FLAG‐Trap agarose beads (Beyotime) for 2 h at 4°C. The beads were washed at least three times with Tris‐buffered saline, and then used for WB analyses.

### 4.10 Subcellular Localisation

The eGFP:ClBeclin1, eGFP:ClBeclin1^∆CC^, ClAPX1:BFP, pCV‐BFP, pART27‐GFP vectors were individually transformed into *A. tumefaciens* GV3101 (pSoup) and then co‐infiltrated with agrobacterial cells carrying the cytoplasmic and nuclear marker mCherry in *N. benthamiana* leaves. *A. tumefaciens* eGFP:ClBeclin1 and ClAPX1:BFP co‐infiltration was also performed on *N. benthamiana* leaves. After 48 h, fluorescence images of the epidermal cells in infiltrated *N. benthamiana* leaves were captured using a scanning confocal microscope (FV3000; Olympus). For eGFP, the excitation wavelength was 488 nm; for mCherry, fluorescence was acquired at 552 nm; for BFP, the excitation wavelength was 400 nm (Du et al. 2023).

### 4.11 Measurement of Ascorbate Peroxidase Activity, JA, MDA, H_2_O_2_ and O^2^ ^−^ Concentration

APX activities were measured using the plant APX assay kit (Solarbio) according to the protocols provided in the manufacturer's instructions. The levels of JA in samples (0.2 g) from citrus roots of pNmGFPer:00, OE‐*ClBeclin1* and RNAi‐*ClBeclin1* were measured by Bonoheng Biotechnology Co. Three biological replicates were used. The JA content in Eureka lemon leaves was determined using the JA ELISA kit (Gelatins). The MDA, H_2_O_2_ and O^2−^ concentrations in OE‐ClBeclin1 or RNAi‐ClBeclin1 citrus hairy roots infected‐CYVCV or mock were detected using specific kits (Solarbio).

### 4.12 Histochemical Staining of DAB and NBT

Citrus hairy roots were collected and stained with NBT or DAB solutions, subjected to negative pressure treatment at −0.1 MPa for 30 min, and then incubated at room temperature in the dark for 12 h. Images were captured using an Olympus DP26 microscope imaging system, and the relative intensity of NBT and DAB staining was measured using ImageJ software (http://rsbweb.nih.gov/ij; Sun and Folimonova 2019).

### 4.13 MeJA, SHAM and H_2_O_2_ Treatment

H_2_O_2_ (Sigma) was dissolved in double‐distilled water, prepared and diluted to 5, 10, 50, 100 and 500 mM concentrations. The dilutions and 100 μM MeJA (Solarbio) and SHAM (Merck) were sprayed onto young Eureka lemon leaves inoculated with CYVCV at 15 dpi. Treatment was repeated every 3 days for a total of three times. Then samples were collected at day 15 for subsequent experiments.

## Conflicts of Interest

The authors declare no conflicts of interest.

## Supporting information


**Figure S1.** Characterisation and expression profile of ClBeclin1. (a) Pairwise identity and conserved domain analysis of Beclin1 from different citrus species, with GenBank accession numbers and citrus species names shown on the left. The Belin1 protein sequences were obtained from *Citrus limon* (Cl), *Atalantia buxfoliata* (Ab), *Citrus australasica* (Ca), *Citrus clementina* (Cc), *Citrus ichangensis* (Ci), *Citrus medica* (Cm), *Citrus reticulata* (Cr), *Citrus sinensis* (Cs), *Fortunella hindsii* (Fh) and *Poncirus trifoliata* (Pt). (b) Phylogenetic analyses of Beclin1. Protein sequences from different plant species were aligned and used to generate a neighbour‐joining phylogenetic tree with 1000 bootstrap replicates in MEGA 11.0 software. (c) Expression of *ClBeclin1* in roots, young leaves, old leaves, young stems and old stems. Values are means ± *SD* (*n* = 3, individual separate plants). Statistical analysis was performed by Student’s *t* test (**p < 0.01, ***p < 0.001). *CsActin* was used as a reference gene.

**Figure S2.** Analysis of the key interaction regions and subcellular localisation of ClBeclin1 and ClAPX1. (a) Yeast two‐hybrid assay revealed the interaction between ClBeclin1 and ClAPXs in yeast. (b) Schematic representation of the three ClBeclin1 truncated mutants. (c) Yeast two‐hybrid assay revealed the interaction of ClBeclin1‐N^1‐167^, ClBeclin1‐CC^168‐296^ and ClBeclin1‐BARA^297‐515^ with ClAPX1 in yeast. (d) The interaction between ClBeclin1‐CC^168‐296^ and ClAPX1 was demonstrated in a bimolecular fluorescence complementation assay. (e) ClBeclin1‐CC^168‐296^ is localised to the cytoplasm. mCherry‐PM (CD3‐1007‐RFP) was used as plasma membrane indicator. Scale bar = 50 μm. The experiment included three technical replicates and 10 fields of view per plant were observed with similar results.

**Figure S3.** Subcellular location of the ClBeclin1‐CC^168‐296^ domain. (a) Subcellular localisation of CC^168‐296^:eGFP and eGFP empty vector control was observed in *Nicotiana benthamiana* after 48 h. mCherry‐H2B and mCherry‐PM (CD3‐1007‐RFP) were used as nuclear and plasma membrane indicators, respectively. The experiment included at least three technical replicates and at least 10 fields of view per plant were observed with similar results. Scale bar = 50 μm. (b) Total protein (T), cytoplasmic protein (C) and cytoplasmic membrane protein (CM) western blot analysis. Anti‐HA and anti‐CsActin antibodies were used in the western blot assay.

**Figure S4.** The effect of ClBeclin1 on the enzymatic activity of ClAPXs and CYVCV coat protein (CP). (a) and (b) ClBeclin1 targets autophagic degradation of CYVCV CP protein via ClAPX1. ClBeclin1:HA and CP were co‐infiltrated into Eureka lemon leaves in the presence or absence of ClAPX1:FLAG. Treatment with the autophagy inhibitor E64D or DMSO in infiltrated Eureka lemon leaves at 12 h post‐inoculation (hpi) and determination of the expression levels of each protein at 48 hpi. (c) and (d) The effect of ClBeclin1 on the enzymatic activity of ClAPX1‐7. ClBeclin1:HA and GUS:FLAG or with ClAPX1‐7:FLAG were transiently co‐expressed in Eureka lemon leaves, and the respective protein expression levels and enzyme activities were determined at 48 hpi. Anti‐CP, anti‐FLAG, anti‐HA and anti‐CsActin antibodies were used in the western blot assay. Asterisks indicate significant differences by Student’s *t* test (*p < 0.05, **p < 0.01, ns, not significant). Values are mean ± *SD* (*n* = 3 technical replicates). All experiments were repeated three times independently.

**Figure S5.** Application of exogenous H_2_O_2_ to Eureka lemons can inhibit CYVCV infection. (a) Phenotype of Eureka lemon plants pretreated with different concentration gradients of H_2_O_2_ (5, 10, 50, 100, 500 mM). (b) Phenotypic symptoms after treatment of CYVCV‐infected Eureka lemon leaves with water (CK) and 50 mM H_2_O_2_. Treatments were made every 3 days for 15 days. Photographs were taken at 15 days post‐inoculation. (c) and (d) The RNA or protein level of CYVCV accumulation in citrus plants after treatment with water (CK) and 50 mM H_2_O_2_. Values are means ± SD (*n* = 3, individual leaves from separate plants). Statistical analysis was performed by Student’s *t* test (**p < 0.01). Anti‐coat protein (CP) and anti‐CsActin antibodies were used in the western blot assay.

**Table S1.** Sequence of primers used in this study.

## Data Availability

The data that support the findings of this study are available from the corresponding author upon reasonable request.
