# Supplementary figures and images for: ClBeclin1 Positively Regulates Citrus Defence Against Citrus Yellow Vein Clearing Virus Through Mediating Autophagy‐Dependent Degradation of ClAPX1
Source: Mol Plant Pathol. 2024 Dec 10;25(12):e70041. doi: 10.1111/mpp.70041 (PMC11631719; doi:10.1111/mpp.70041)

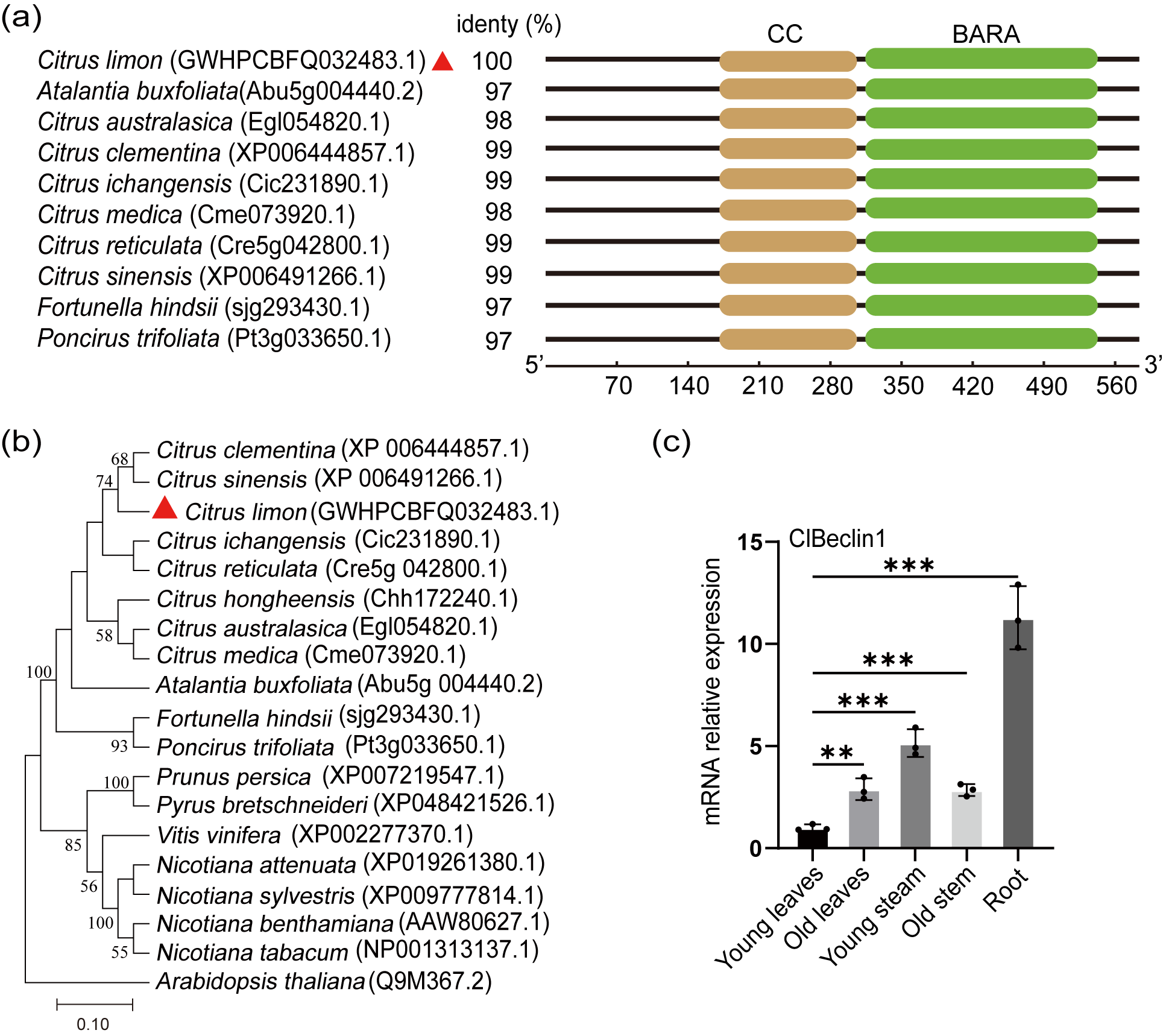

Supplement: Supplementary file 1 — Figure S1. Characterisation and expression profile of ClBeclin1. (a) Pairwise identity and conserved domain analysis of Beclin1 from different citrus species, with GenBank accession numbers and citrus species names shown on the left. The Belin1 protein sequences were obtained from Citrus limon (Cl), Atalantia buxfoliata (Ab), Citrus australasica (Ca), Citrus clementina (Cc), Citrus ichangensis (Ci), Citrus medica (Cm), Citrus reticulata (Cr), Citrus sinensis (Cs), Fortunella hindsii (Fh) and Poncirus trifoliata (Pt). (b) Phylogenetic analyses of Beclin1. Protein sequences from different plant species were aligned and used to generate a neighbour‐joining phylogenetic tree with 1000 bootstrap replicates in MEGA 11.0 software. (c) Expression of ClBeclin1 in roots, young leaves, old leaves, young stems and old stems. Values are means ± SD (n = 3, individual separate plants). Statistical analysis was performed by Student’s t test (**p < 0.01, ***p < 0.001). CsActin was used as a reference gene. [file MPP-25-e70041-s001.tif]

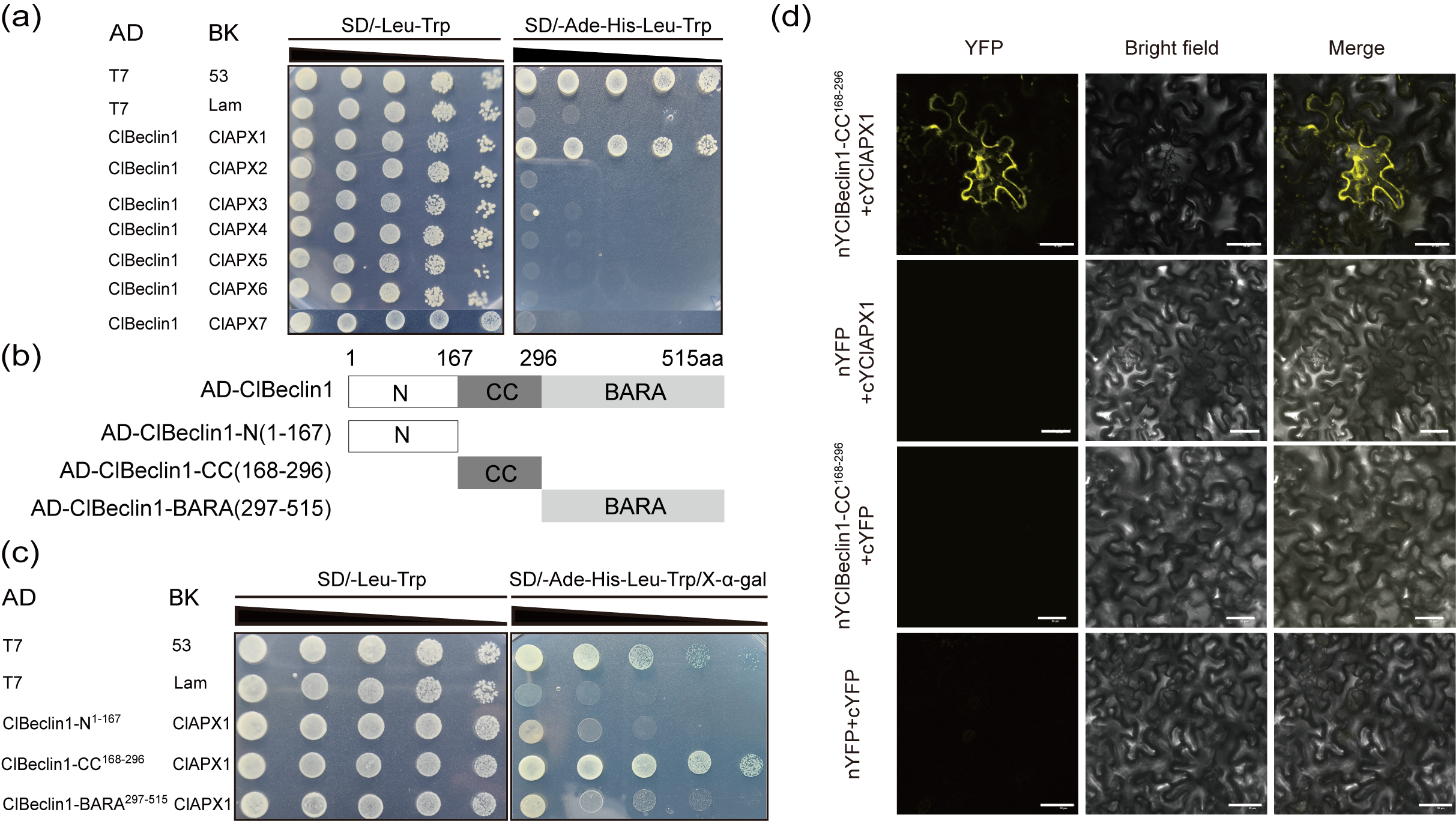

Supplement: Supplementary file 2 — Figure S2. Analysis of the key interaction regions and subcellular localisation of ClBeclin1 and ClAPX1. (a) Yeast two‐hybrid assay revealed the interaction between ClBeclin1 and ClAPXs in yeast. (b) Schematic representation of the three ClBeclin1 truncated mutants. (c) Yeast two‐hybrid assay revealed the interaction of ClBeclin1‐N1‐167, ClBeclin1‐CC168‐296 and ClBeclin1‐BARA297‐515 with ClAPX1 in yeast. (d) The interaction between ClBeclin1‐CC168‐296 and ClAPX1 was demonstrated in a bimolecular fluorescence complementation assay. (e) ClBeclin1‐CC168‐296 is localised to the cytoplasm. mCherry‐PM (CD3‐1007‐RFP) was used as plasma membrane indicator. Scale bar = 50 μm. The experiment included three technical replicates and 10 fields of view per plant were observed with similar results. [file MPP-25-e70041-s006.tif]

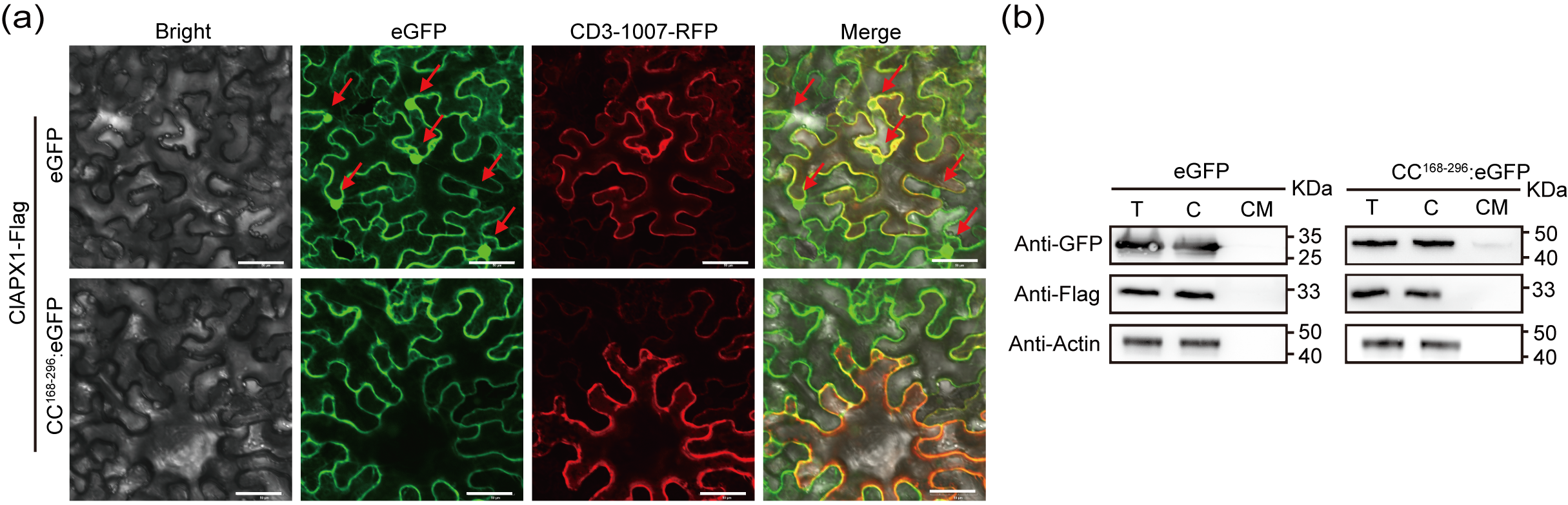

Supplement: Supplementary file 3 — Figure S3. Subcellular location of the ClBeclin1‐CC168‐296 domain. (a) Subcellular localisation of CC168‐296:eGFP and eGFP empty vector control was observed in Nicotiana benthamiana after 48 h. mCherry‐H2B and mCherry‐PM (CD3‐1007‐RFP) were used as nuclear and plasma membrane indicators, respectively. The experiment included at least three technical replicates and at least 10 fields of view per plant were observed with similar results. Scale bar = 50 μm. (b) Total protein (T), cytoplasmic protein (C) and cytoplasmic membrane protein (CM) western blot analysis. Anti‐HA and anti‐CsActin antibodies were used in the western blot assay. [file MPP-25-e70041-s005.tif]

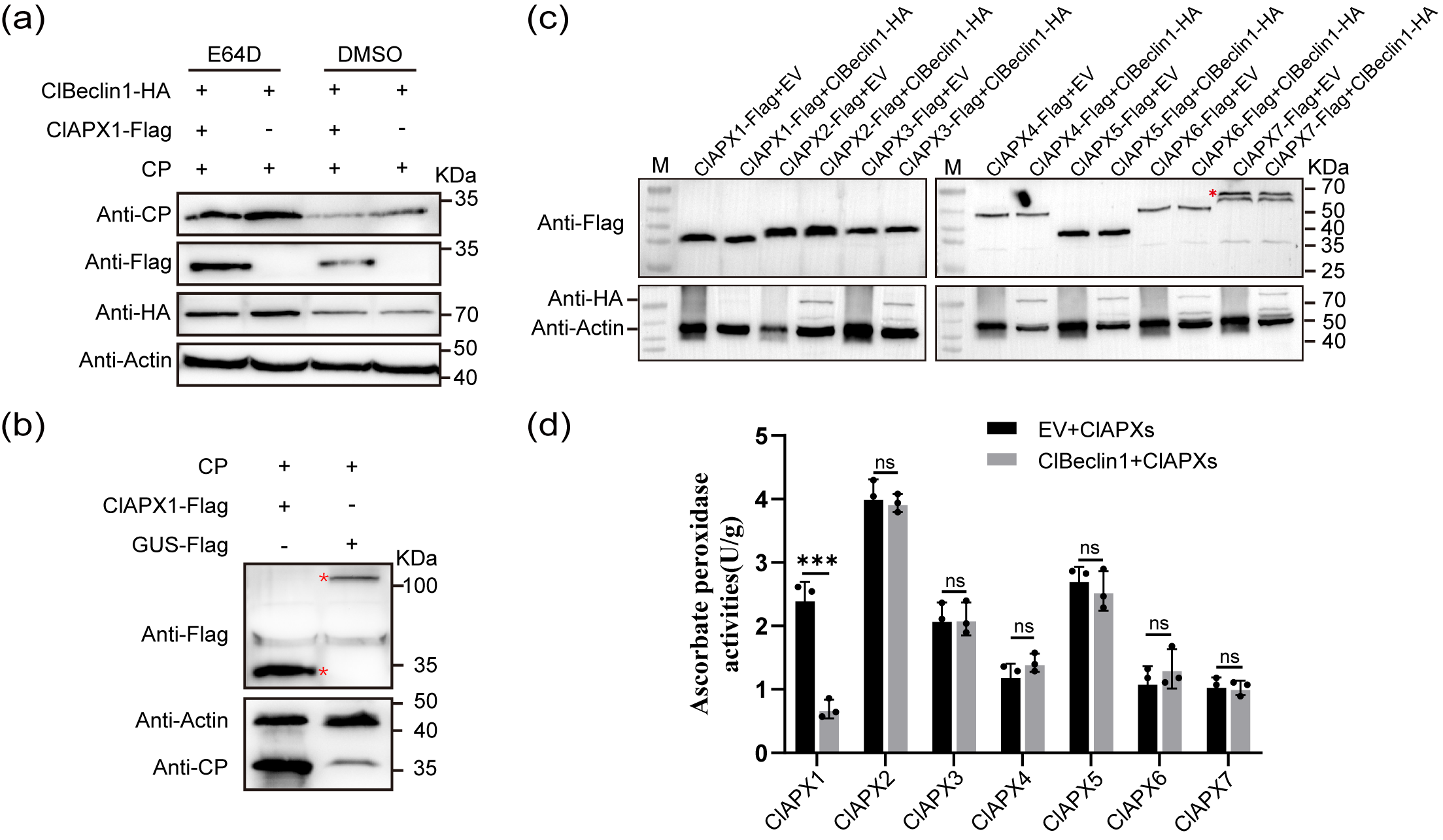

Supplement: Supplementary file 4 — Figure S4. The effect of ClBeclin1 on the enzymatic activity of ClAPXs and CYVCV coat protein (CP). (a) and (b) ClBeclin1 targets autophagic degradation of CYVCV CP protein via ClAPX1. ClBeclin1:HA and CP were co‐infiltrated into Eureka lemon leaves in the presence or absence of ClAPX1:FLAG. Treatment with the autophagy inhibitor E64D or DMSO in infiltrated Eureka lemon leaves at 12 h post‐inoculation (hpi) and determination of the expression levels of each protein at 48 hpi. (c) and (d) The effect of ClBeclin1 on the enzymatic activity of ClAPX1‐7. ClBeclin1:HA and GUS:FLAG or with ClAPX1‐7:FLAG were transiently co‐expressed in Eureka lemon leaves, and the respective protein expression levels and enzyme activities were determined at 48 hpi. Anti‐CP, anti‐FLAG, anti‐HA and anti‐CsActin antibodies were used in the western blot assay. Asterisks indicate significant differences by Student’s t test (*p < 0.05, **p < 0.01, ns, not significant). Values are mean ± SD (n = 3 technical replicates). All experiments were repeated three times independently. [file MPP-25-e70041-s002.tif]

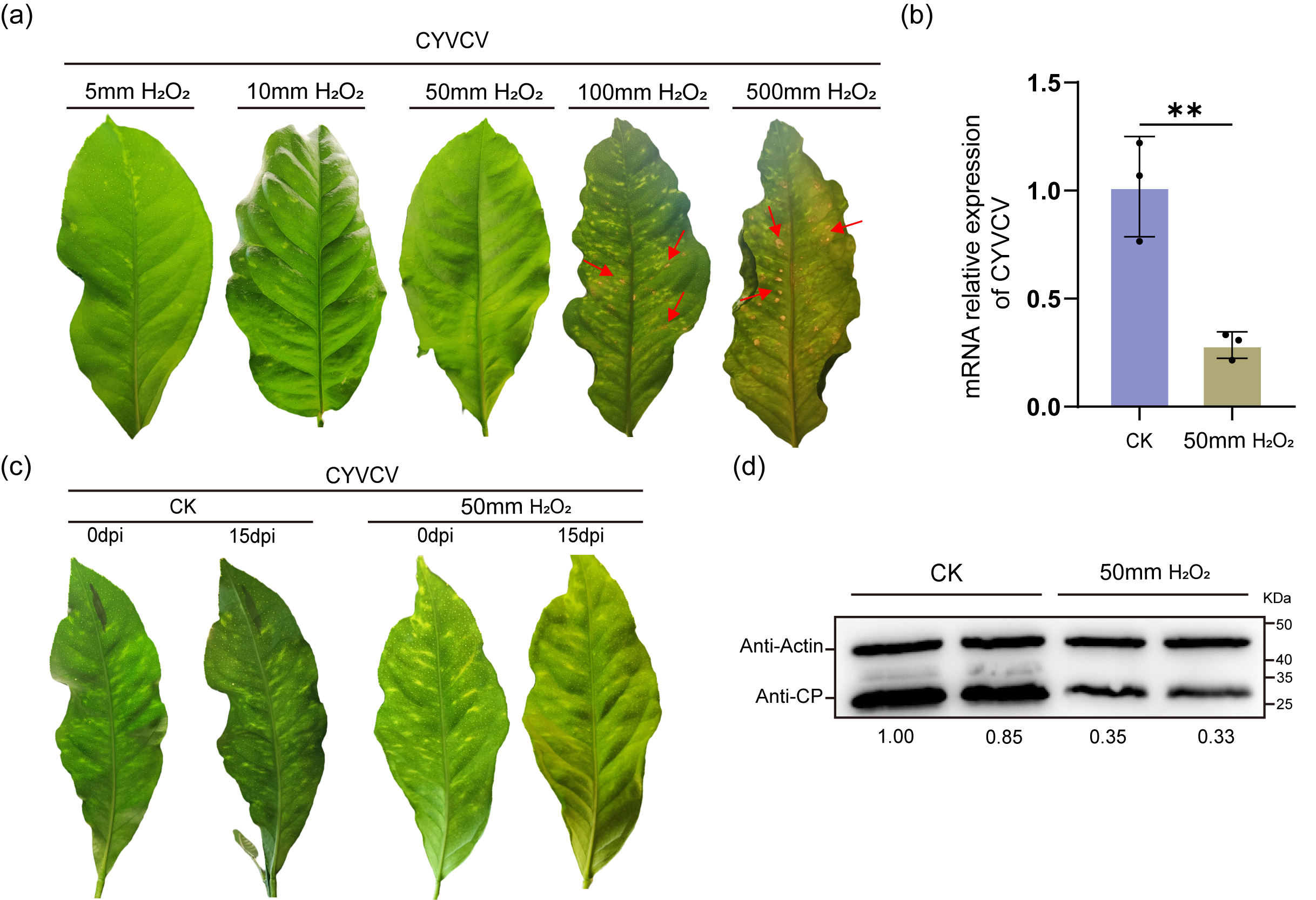

Supplement: Supplementary file 5 — Figure S5. Application of exogenous H2O2 to Eureka lemons can inhibit CYVCV infection. (a) Phenotype of Eureka lemon plants pretreated with different concentration gradients of H2O2 (5, 10, 50, 100, 500 mM). (b) Phenotypic symptoms after treatment of CYVCV‐infected Eureka lemon leaves with water (CK) and 50 mM H2O2. Treatments were made every 3 days for 15 days. Photographs were taken at 15 days post‐inoculation. (c) and (d) The RNA or protein level of CYVCV accumulation in citrus plants after treatment with water (CK) and 50 mM H2O2. Values are means ± SD (n = 3, individual leaves from separate plants). Statistical analysis was performed by Student’s t test (**p < 0.01). Anti‐coat protein (CP) and anti‐CsActin antibodies were used in the western blot assay. [file MPP-25-e70041-s004.tif]
