# Supplementary material for: ClBeclin1 Positively Regulates Citrus Defence Against Citrus Yellow Vein Clearing Virus Through Mediating Autophagy‐Dependent Degradation of ClAPX1
Source: Mol Plant Pathol. 2024 Dec 10;25(12):e70041. doi: 10.1111/mpp.70041 (PMC11631719; doi:10.1111/mpp.70041)
Supplement: Supplementary file 6 — Table S1. Sequence of primers used in this study. [file MPP-25-e70041-s003.docx]

Table S1 Sequence of primers used in this study

| Primer name | Primer sequence (5＇-3＇) |
| --- | --- |
| **PCR assay** |  |
| pNmGFPer-GFP-JJ-F | GGCACAAATTTTCTGTAAGT |
| pNmGFPer-GFP-JJ-R | ACAGGGCCATCGCC |
| pNmGFPer-ClBeclin1-JJ-F | TCGTTGAAGATGCCTCTGCCGACAG |
| pNmGFPer-ClBeclin1-JJ-R | CTTTCCAGACTGACTCGCCTCAG |
| **RT-qPCR** |  |
| RT-CsActin-F | CATCCCTCAGCACCTTCC |
| RT-CsActin-R | CCAACCTTAGCACTTCTCC |
| RT-CYVCV-F | TCAACCTCAACCTAAGATA |
| RT-CYVCV-R | GATGGCGTATAAGAACAC |
| RT-ClBeclin1-F | GTACCTCGATGGGTTTGCCA |
| RT-ClBeclin1-R | ACACTGTTGGACGCATGGAT |
| RT-ClAPX1-F | TTGTTGCTCTCTCTGGTGGC |
| RT-ClAPX1-R | CAGTCAAGAGCTCCGTGAAGT |
| RT-CsMCA1-F | GCTAACCACACCATAACG |
| RT-CsMCA1-R | TTGTATCATTATATGTTACTCCAC |
| RT-CsHSR203-F | ACGCGTCTTCCTCATTGGAG |
| RT-CsHSR203-R | ACCCGGATGAATCGGAATCG |
| RT-AOX1a-F | AACTGCTTTAGGCCATGGGA |
| RT-AOX1a-R | ATCCGAAAAGGTCGTCGGTG |
| RT-CsOPR3-F | GGCTCAAACTTCACATGAAACTAC |
| RT-CsOPR3-R | GTTGACAATAATCTCCAGGGTTTC |
| RT-CsLOX2-F | GTCGTTCTGGAACTTGTCGGC |
| RT-CsLOX2-R | CTGTGATTGCACCAGGCGT |
| RT-CsAOS1-2-F | CCACCTACACGGAGGCATTT |
| RT-CsAOS1-2-R | CGGGGTTCTTACCGAACCAA |
| RT-CsAUX-F | GGTCTTTCCTTGGCCTTGGT |
| RT-CsAUX-R | AGTCACACCATCAACCTGGC |
| RT-CsHSP90-F | AGCAAGCTAGATGCCCAACC |
| RT-CsHSP90-R | AGGTTGTTCACCAAATCAGCC |
| RT-CsERO2-F | TTGCCCTACATATCCATCCAAACA |
| RT-CsERO2-R | TGTGAGATTTTGACCCCACAAA |
| RT-CsSOD-F | GTGGTGGTGGAAAGCCATCT |
| RT-CsSOD-R | GTCTATTGGCTTTGTATGCCAG |
| RT-CsP450-F | TGTAGCTGCTGTGGGGAAAG |
| RT-CsP450-R | AGAAAATGGCCAGGAGGGTG |
| RT-CsRBOHA-F | TCCATTCGAGTGGCATCCAT |
| RT-CsRBOHA-R | AGCCCACTTTTCCCAGCTAC |
| RT-CsASA-F | CATTTTGTTCCCGCAGTGCT |
| RT-CsASA-R | TGCCCACTAATCTCCCAAGC |
| RT-CsGST-F | TAAACTCCCGTTGATGCCCTC |
| RT-CsGST-R | CTCCTTCCACAATCGTAAATCTTC |
| **Transient expression assay** |  |
| pNmGFPer-ClAPX1-Flag-F | TTTGGAGAGGACAGGGTACCATGACGAAGAATTACCCCACTGTTAGC |
| pNmGFPer-ClAPX1-Flag-R | ATCCTCCTCCGGATCCGGCTTCAGCAAATCCTAGCTCAG |
| pNmGFPer-ClAPX2-Flag-F | TTTGGAGAGGACAGGGTACCATGGGAAAGTGTTATCCAAAAGTGAGC |
| pNmGFPer-ClAPX2-Flag-R | ATCCTCCTCCGGATCCCTCAGCATCCGCAAATCCGAG |
| pNmGFPer-ClAPX3-Flag-F | TTTGGAGAGGACAGGGTACCATGGCTTTACCGGTCGTTGAC |
| pNmGFPer-ClAPX3-Flag-R | ATCCTCCTCCGGATCCCTTCATCCTTTTGCGAACTTCGTAAAAGT |
| pNmGFPer-ClAPX4-Flag-F | TTTGGAGAGGACAGGGTACCATGGGCGCTTCTTTTCTCTCTAC |
| pNmGFPer-ClAPX4-Flag-R | ATCCTCCTCCGGATCCCAATTTGAGTTTGCTCAATTCAATCTTTTGAGC |
| pNmGFPer-ClAPX5-Flag-F | TTTGGAGAGGACAGGGTACCATGGTGGGAAGCATCGCAAT |
| pNmGFPer-ClAPX5-Flag-R | ATCCTCCTCCGGATCCCTTGGTTTTTCTGTGAACTTCATAAAAGTAACTGAAAA |
| pNmGFPer-ClAPX6-Flag-F | TTTGGAGAGGACAGGGTACCATGAGTTCATCAACTGCCTCCAG |
| pNmGFPer-ClAPX6-Flag-R | ATCCTCCTCCGGATCCCAAGCTTCTCCACCTTGCTCC |
| pNmGFPer-ClAPX7-Flag-F | TTTGGAGAGGACAGGGTACCATGGCAGCTCGGCCAG |
| pNmGFPer-ClAPX7-Flag-R | ATCCTCCTCCGGATCCGTCCTTTCCGGACGAATACTTGG |
| pNmGFPer-GUS-Flag-F | TTTGGAGAGGACGGGTACCATGTTACGTCCTGTAGAAACCCCA |
| pNmGFPer-GUS-Flag-R | ATCCTCCTCCGGATCCGAAGAACTCGTCAAGAAGGCGATAGAAG |
| pNmGFPer-CP -F | TTTGGAGAGGACAGGGTACCATGAGCTTCGACTACACTCACCC |
| pNmGFPer-CP-R | ATTCCTGCAGGTCGACGATGTTGAAAGGGGTCGGGC |
| pART27-eGFP-ClBeclin1-F1 | AGAGGACACGCTCGAGATGAAGAAAGAGGACGCACCG |
| pART27-eGFP-ClBeclin1-R2 | TGCTCACCATCTCGAGTGGCTTTGACAAATTTCGACCAACAGATTTGAGGT |
| pART27-eGFP-ClBeclin1^∆CC^-F2 | TGAGCAAGAGTTGTTGAAGCGAACTAATGTACTTAATG |
| pART27-eGFP-ClBeclin1^∆CC^-R1 | AACAACTCTTGCTCAACCTGTGTCTGGGATG |
| **Transgenic hairy roots** |  |
| pNmGFPer-ClBeclin1-F | TTTGGAGAGGACAGGGTACCATGAAGAAAGAGGACGCACCG |
| pNmGFPer-ClBeclin1-R | ATTCCTGCAGGTCGACTGGCTTTGACAAATTTCGACCAACAG |
| pNmGFPer-RNAi- ClBeclin1（+）F | ATCCCATGGGGCGCGCCATGAAGAAAGAGGACGCACCG |
| pNmGFPer-RNAi- ClBeclin1（+）R | AATTCTTACACATTTAAATACTCGCCTCAGATTGGGC |
| pNmGFPer-RNAi- ClBeclin1（-）F | GGATCCTAGGTGAGTCTAGACTCGCCTCAGATTGGGC |
| pNmGFPer-RNAi- ClBeclin1（-）R | TCCTGCAGGTCGACTCTAGAATGAAGAAAGAGGACGCACCG |
| **Subcellular localization** |  |
| pART27-eGFP-ClBeclin1-F | AGAGGACACGCTCGAGATGAAGAAAGAGGACGCACCG |
| pART27-eGFP-ClBeclin1-R | TGCTCACCATCTCGAGTGGCTTTGACAAATTTCGACCAACAGATTTGAGGT |
| PCV-ClAPX1-BFP -F: | TGAAGCCATGGTGAGCAAGGGCGAG |
| PCV-ClAPX1-BFP -R: | ATCGGGGAAATTCGAGCTCCGATCCTCCTCCCTTGTACAGCTCGTCCATGCC |
| pART27-eGFP-ClBeclin1-CC-F | AGAGGACACGCTCGAGATGCCTTTATGTCTCGAGTGCATGAGG |
| pART27-eGFP-ClBeclin1-CC-R | TGCTCACCATCTCGAGTAAATGTGCTTGTGAAACTTCTATCTTAGACGAAATTGC |
| **Interaction validation** |  |
| pGADT7-ClBeclin1-F | GGAGGCCAGTGAATTCATGAAGAAAGAGGACGCACCG |
| pGADT7-ClBeclin1-R | CGAGCTCGATGGATCCTCATGGCTTTGACAAATTTCGACCAACAG |
| pGADT7-ClBeclin1-N-F | GGAGGCCAGTGAATTCATGAAGAAAGAGGACGCACCG |
| pGADT7-ClBeclin1-N-R | CGAGCTCGATGGATCCTTGCTCAACCTGTGTCTGGGAT |
| pGADT7-ClBeclin1-CC-F | GGAGGCCAGTGAATTCATGCCTTTATGTCTCGAGTGCATGAGG |
| pGADT7-ClBeclin1-CC-R | CGAGCTCGATGGATCCTAAATGTGCTTGTGAAACTTCTATCTTAGACG |
| pGADT7-ClBeclin1-BARA-F | GGAGGCCAGTGAATTCATGGAGTTGTTGAAGCGAACTAATGTACTTAATGA |
| pGADT7-ClBeclin1-BARA-R | CGAGCTCGATGGATCCTGGCTTTGACAAATTTCGACCAACAGAT |
| pCV-clUC-ClBeclin1-F | ACGGGGGACGAGCTCGGTACCATGAAGAAAGAGGACGCACCG |
| pCV-clUC-ClBeclin1-R | CGCGTACGAGATCTGGTCGACTGGCTTTGACAAATTTCGACCAACAG |
| pCV-nYFP-ClBeclin1-F | AACATCGAGGACTCCGGAGTCGACATGAAGAAAGAGGACGCACCG |
| pCV-nYFP-ClBeclin1-R | GAATTCGAGCTCGCCTGGGGATCCTGGCTTTGACAAATTTCGACCAACAG |
| pCV-nYFP-ClBeclin1-N-F | AACATCGAGGACTCCGGAGTCGACATGAAGAAAGAGGACGCACCG |
| pCV-nYFP-ClBeclin1-N-R | GAATTCGAGCTCGCCTGGGGATCCTGCTCAACCTGTGTCTGGGAT |
| pCV-nYFP-ClBeclin1-CC-F | AACATCGAGGACTCCGGAGTCGACATGCCTTTATGTCTCGAGTGCATGAGG |
| pCV-nYFP-ClBeclin1-CC-R | GAATTCGAGCTCGCCTGGGGATCCAAATGTGCTTGTGAAACTTCTATCTTAGACG |
| pCV-nYFP-ClBeclin1-BARA-F | AACATCGAGGACTCCGGAGTCGACATGGAGTTGTTGAAGCGAACTAATGTACTTAATGA |
| pCV-nYFP-ClBeclin1-BARA-R | GAATTCGAGCTCGCCTGGGGATCCGGCTTTGACAAATTTCGACCAACAGAT |
| pGBKT7-ClAPX1-F | CATGGAGGCCGAATTCATGACGAAGAATTACCCCACTGTTAGC |
| pGBKT7-ClAPX1-R | GCCGCTGCAGGTCGACGGCTTCAGCAAATCCTAGCTCAGAG |
| pCV-nlUC-ClAPX1-F | ACGGGGGACGAGCTCGGTACCATGACGAAGAATTACCCCACTGTTAGC |
| pCV-nlUC-ClAPX1-R | AACATCGTATGGGTAGTCGACGGCTTCAGCAAATCCTAGCTCAG |
| pCV-cYFP-ClAPX1-F | GAGCTGTACAAGTCCGGAGTCGACATGACGAAGAATTACCCCACTGTTAGC |
| pCV-cYFP-ClAPX1-R | GAATTCGAGCTCGCCTGGGGATCCGGCTTCAGCAAATCCTAGCTCAG |
